# Lemon basil seed-derived peptide: Hydrolysis, purification, and its role as a pancreatic lipase inhibitor that reduces adipogenesis by downregulating SREBP-1c and PPAR-γ in 3T3-L1 adipocytes

**DOI:** 10.1371/journal.pone.0301966

**Published:** 2024-05-22

**Authors:** Kittisak Kuptawach, Sajee Noitung, Anumart Buakeaw, Songchan Puthong, Ruengwit Sawangkeaw, Papassara Sangtanoo, Piroonporn Srimongkol, Onrapak Reamtong, Kiattawee Choowongkomon, Aphichart Karnchanatat

**Affiliations:** 1 Program in Biotechnology, Faculty of Science, Chulalongkorn University, Bangkok, Thailand; 2 Center of Excellence in Bioconversion and Bioseparation for Platform Chemical Production, Institute of Biotechnology and Genetic Engineering, Chulalongkorn University, Bangkok, Thailand; 3 Department of Molecular Tropical Medicine and Genetics, Faculty of Tropical Medicine, Mahidol University, Bangkok, Thailand; 4 Department of Biochemistry, Faculty of Science, Kasetsart University, Bangkok, Thailand; Faculty of Sciences and Technology, State Islamic University of Sunan Kalijaga, INDONESIA

## Abstract

The purpose of this study is to assess the bioactive peptides derived from the defatted lemon basil seeds hydrolysate (DLSH) for their ability to inhibit pancreatic lipase, decrease intracellular lipid accumulation, and reduce adipogenesis. Response surface methodology (RSM) was employed to optimize trypsin hydrolysis conditions for maximizing lipase inhibitory activity (LI). A hydrolysis time of 387.06 min, a temperature of 49.03°C, and an enzyme concentration of 1.61% w/v, resulted in the highest LI with an IC_50_ of 368.07 μg/mL. The ultrafiltration of the protein hydrolysate revealed that the fraction below 0.65kDa exhibited the greatest LI potential. Further purification *via* RP-HPLC identified the Gly-Arg-Ser-Pro-Asp-Thr-His-Ser-Gly (GRSPDTHSG) peptide in the HPLC fraction F_1_ using mass spectrometry. The peptide was synthesized and demonstrated LI with an IC_50_ of 0.255 mM through a non-competitive mechanism, with a constant (K_i_) of 0.61 mM. Docking studies revealed its binding site with the pancreatic lipase-colipase complex. Additionally, GRSPDTHSG inhibited lipid accumulation in 3T3-L1 cells in a dose-dependent manner without cytotoxic effects. Western blot analysis indicated downregulation of PPAR-γ and SREBP-1c levels under GRSPDTHSG treatment, while an increase in AMPK-α phosphorylation was observed, suggesting a role in regulating cellular lipid metabolism. Overall, GRSPDTHSG demonstrates potential in attenuating lipid absorption and adipogenesis, suggesting a prospective application in functional foods and nutraceuticals.

## Introduction

Obesity and hyperlipidemia, two interrelated metabolic disorders, greatly affect human well-being and pose significant challenges to global public health. Obesity occurs when adipose tissue depots expand beyond their normal storage capacity, resulting in a body mass that exceeds healthy weight ranges. Hyperlipidemia indicates elevated levels of plasma lipids, such as cholesterol and triglycerides. These conditions often coexist, heightening the likelihood of several complications [1, 2]. Obesity directly contributes to hyperlipidemia by altering lipid metabolism. The excessive adipose tissue leads to increased production of free fatty acids, which can eventually stimulate insulin resistance and dyslipidemia [3]. Hyperlipidemia serves as a risk factor for the initiation of atherosclerosis by causing the accumulation of plaque in the artery walls, which can lead to heart attacks and strokes [4]. Furthermore, they are linked to certain types of cancer, such as breast, colon, gallbladder, pancreas, uterus, and ovary [5]. Additionally, they can contribute to the development of non-alcoholic fatty liver disease, potentially leading to more serious liver issues [6]. It is understood that the limitation of calorie intakes and the promotion of physical exercise should lead to improvements in health, but this approach does not inevitably lead to success. Further factors, including the extent of the obesity level and the capacity of the individual to adhere to the healthy lifestyle regimen will influence the likelihood of successful weight loss. Where behavioral change fails, more extreme measures such as medication to induce weight loss or even surgery may be attempted [7, 8].

In the pursuit of effective methods to manage obesity, researchers are increasingly focusing on strategies aimed at manipulating key biochemical pathways involved in lipid absorption and accumulation. Among these promising avenues are the inhibition of lipase activity and adipogenesis, each targeting distinct stages within the lipid metabolism process [9]. Lipases are enzymes responsible for the breakdown of dietary triglycerides into absorbable forms of fatty acids and glycerol in the digestive tract. Therefore, inhibiting lipase activity can reduce the amount of lipid absorption and limit the availability of substrates for adipogenesis. This results in reduced calorie intake, facilitating weight loss endeavors [10]. Concurrently, adipogenesis is the process through which preadipocytes develop into mature adipocytes or fat cells. This process involves several phases, including cell proliferation, differentiation into mature cells, and lipid accumulation. Mature adipocytes function to store excess energy in the form of lipids or triglycerides. Inhibiting adipogenesis can mitigate the formation of new adipocytes, thus restricting overall lipid accumulation in the body [11, 12]. Many studies have considered the possibility of using natural compounds to modulate adipocyte differentiation and knowledge in this field may be further extended by findings that a novel peptide can demonstrate anti-obesity properties. The creation and subsequent expansion of adipose tissue is significantly influenced by adipogenesis, which is the process by which preadipocytes differentiate to produce mature adipocytes. This process of adipocyte differentiation can be demonstrated *in vitro*, using 3T3-L1 cells, preadipocytes, or matrix-derived precursor cells obtained from adipose tissue [13, 14]. The differentiation of adipocytes is a process initiated by various signaling pathways, and transcription factors induce preadipocytes to form mature adipocytes. The transition is regulated by the main adipogenic transcription factors which include peroxisome proliferator-activated receptor gamma (PPAR-γ) and CCAAT/enhancer binding protein alpha (C/EBP-α). These transcription factors are highly expressed when differentiation takes place, and they combine to further induce the downstream expression of adipose-specific genes such as resistin, adiponectin, and adipocyte fatty acid-binding protein 2 (aP2). Furthermore, sterol regulatory element-binding protein-1c (SREBP-1c) can further promote the activity of PPAR-γ when adipocyte differentiation takes place. The transcription factors are also able to play roles in the activation of enzymes related to metabolism such as acetyl-CoA carboxylase (ACC) or fatty acid synthase (FAS). Meanwhile, adenosine monophosphate-activated protein kinase (AMPK), responds to energy status and can restrict the synthesis of lipids through the inhibition of ACC protein activity when activated. In the liver, activated AMPK phosphorylates works within the liver to deactivate the enzymes which contribute to lipogenesis, one of which is ACC, thus confirming the importance of AMPK/ACC signaling during the process of hepatic lipid homeostasis [15–17].

Obesity and hyperlipidemia were managed through the use of synthetic drugs such as Sibutramine (Reductil) [18], which suppresses appetite, or Orlistat (Xenical®) [19], which inhibits pancreatic lipase, thereby limiting the absorption of fat in the small intestines. However, the adverse side effects are numerous and problematic, including such issues as allergic reactions (hives), swelling of the face, tongue, and throat, gastrointestinal or rectal pain, urgent or oily bowel movements, breathing problems, nausea, diarrhea, and vomiting [20, 21]. It is therefore imperative to discover a natural alternative treatment which avoids these side effects while delivering better outcomes at a lower cost. At present, few researchers have adequately explored the field of natural remedies which effectively target the mechanisms underpinning obesity, but food-derived compounds are thought to offer strong potential, with peptides and proteins proving especially interesting [22, 23]. Earlier studies have demonstrated the suitability of rice bran [24], soy [25], and chickpeas [26] as sources of plant-derived peptides and proteins, which exhibit inhibitory effects both *in vitro* and *in vivo*. These peptides demonstrate lipase inhibition and reduction of lipidemia, attributed to specific amino acid sequences formed through the enzymatic hydrolysis of the relevant proteins. As a result, peptides derived from protein hydrolysates can be considered strong candidates for development as functional foods or supplements with few adverse side effects.

Lemon basil (*Ocimum citriodorum*), an herb known for its aromatic and tasty properties, may be a source of high-quality proteins for the isolation of peptides. Notably, a prior study that obtained lemon basil seed oil using supercritical carbon dioxide extraction revealed that it contained a remarkable 90% unsaturated fatty acid content. The protein percentage of the residual product from this extraction procedure is around 57.16%, indicating that defatted lemon basil seed has the potential to serve as a significant source of protein [27]. In previous investigations, two antihypertensive peptides (LGRNLPPI and GPAGPAGL) were obtained from basil seeds hydrolysate (DLSH) through hydrolysis by Alcalase^®^ to inhibit angiotensin I-converting enzyme (ACE) [28]. Additionally, the calcium chelation properties of the isolated peptides AFNRAKSKALNEN (Basil-1) and YDSSGGPTPWLSPY (Basil-2) were investigated using the Caco-2 cell as a model [29]. Expanding on our previous work, this study aims to evaluate DLSH-derived peptides by utilizing trypsin hydrolysis in 3T3-L1 adipocytes in terms of their potential to lipase inhibitory activity (LI), inhibit lipid accumulation, and their anti-adipogenic effects. The development of functional foods or novel supplements based on the bioactive potential of plant-derived peptides can be facilitated by the development of a better understanding of the role of such peptides in modulating lipid metabolism. It is anticipated that this study may provide useful findings in this context.

## Materials and methods

### Chemicals and biomaterials

This study made use of Thai lemon basil seeds sourced from Royal Thai Seeds Co., Ltd., originating from Si Samrong District, Sukhothai Province, from a site located at an altitude of 50 m. Harvesting of the seeds took place in December 2018 when the plants in question were 120 days old. The seeds were then air dried prior to being stored in the dark at a temperature of 4°C. The chemicals and reagents used in this study were all analytical grade. Sigma-Aldrich (St. Louis, MO, USA) provided bovine serum albumin (BSA), Coomassie Brilliant Blue G-250, dimethyl sulfoxide (DMSO), protease inhibitor cocktail P8340, lipase (porcine pancreas), trypsin (porcine pancreas), sodium dodecyl sulfate (SDS), *p*-nitrophenyl palmitate (*p*NPP), o-phthaldehyde (OPA), sodium tetraborate decahydrate, (−)-tetrahydrolipstatin, *N*-Formyl-L-leucine (1S)-1-[[(2S,3S)-3-hexyl-4-oxo-2-oxetanyl]methyl] dodecyl ester (Orlistat), and trifluoroacetic acid (TFA) while dithiothreitol (DTT) and Tris Base were supplied by Merck (Darmstadt, Germany). NP40 cell lysis buffer, primary antibodies including; β-actin (Rabbit polyclonal antibody; PA1-183), AMPK-α (Rabbit monoclonal antibody; SU03-48), PPAR-γ (Rabbit monoclonal antibody; MA5-14889), SREBP-1c (Rabbit polyclonal antibody; PA1-337), Secondary antibody (horseradish peroxidase (HRP)-conjugated goat anti-rabbit IgG) were provided by Thermo Fisher Scientific (San Jose, CA, USA). Horseradish peroxidase (HRP) substrates was supplied by Bio-Helix (New Taipei City, Taiwan). All of the substances utilized in this study were of analytical grade.

### Experimental design

The experimental design for optimization underwent modifications from the previous approach [19, 48]. The initial experimentation focused on single variable effects, providing analysis of the influence on the dependent variables of degree of hydrolysis (DH) and lipase inhibitory activity (LI) of hydrolysis time (30, 60, 120, 180, 240, 300, and 360 min), temperature (30, 40, 50, and 60°C), and enzyme concentration (0.5, 1, 1.5, 2, and 2.5% w/v). With the variation of each factor, the optimal experimental design could be determined *via* response surface methodology (RSM), while the optimal condition prediction was determined by regression analysis using the Design Expert 12 program (Stat-Ease, Inc., USA) with central composite design (CCD). In this case, CCD comprised 20 experiment trials based on five levels for each of the factors of time, temperature, and enzyme concentration, which served as independent variables. These variable levels can be seen in S1 Table. Meanwhile, the role of dependent variables was fulfilled by degree of hydrolysis and lipase inhibition.

Analysis of the experimental data resulted in the formation of a second-order equation describing the effects of the linear, quadratic, and interaction terms of the various factors upon the outcomes for DH and LI. This equation can be seen below as Eq 1.

$$\text{Y}=\beta_{0}+\Sigma \beta_{\text{i}}{\text{x}}_{\text{i}}+\Sigma \beta_{\text{ij}}{\text{x}}_{\text{i}}{\text{x}}_{\text{j}}+\Sigma \beta_{\text{i}}{\text{x}}_{ii}^{2}$$ (1)

in which Y indicates the response value, β_0_ represents the model intercept, the independent variables are given by xi and x_j_, and β_i_, β_ii_, and β_ij_ serve respectively as the linear, quadratic, and interaction coefficients.

### Preparation of the protein hydrolysate

Preparation of the defatted lemon basil seed powder was conducted in line with the approach of Kheeree *et al*. [28]. The powder (5% w/v) was first suspended in 50 mL of 20 mM phosphate buffer saline (PBS) at a pH value of 8.0. Hydrolysis then took place in a shaker incubator with trypsin before the reaction was terminated by heating in water bath at a temperature of 90°C for 20 min. In the final stage, the solution underwent centrifugation at 10,000 rpm for 30 min at 4°C before the supernatant was gathered and placed in storage at -20°C until required.

### Determination of the degree of hydrolysis (DH)

DH can be defined as the cleaved peptide bond percentage. It can be determined *via* the Nielsen method [30] which involves the use of OPA solution. The absorbance of a compound produced when the OPA molecules and the SH-group of DTT reacted with the primary amino groups of the hydrolyzed peptides was measured at 340 nm, with serine employed as a standard. Eq 2 was then used to calculate the degree of hydrolysis.

DH=(h/htot)×100 (2)

in which htot indicates the overall number of peptide bonds per protein equivalent, defined as 8.054, and the total number of hydrolyzed bonds is given by h.

Determination of the protein concentration. A Bradford assay was carried out using BSA (bovine serum albumin) as the standard to assess the protein concentration of DLSH [31]. A calibration curve was plotted before the absorbance of a mixture of 20 μL of the protein sample and 200 μL of Bradford working solution was evaluated at 595 nm. As the amino group reacts with the Coomassie Brilliant Blue G-250 dye, the outcome is a change from the original brown color to blue.

### Evaluation of lipase inhibitory activity

The determination of lipase inhibition was performed using a method with slight modifications as described by Ketprayoon *et al*. [24]. The *p*-nitrophenyl palmitate (*p*NPP) was served as the substrate, with a solution containing 30 mg of *p*NPP dissolved in 10 mL of isopropanol. This pNPP solution was then emulsified by combining it with 90 mL of 50 mM Tris-HCl at pH 7.0, 20 μL of Triton X-100 and 100 mg of gum arabic. The assay was initiate the assay by incubating the porcine pancreatic lipase (0.5 mg/mL in 50 mM Tris‐HCl buffer, pH 7.0), *p*NPP solution and DLSH peptides at a temperature of 37°C for 30 min. Absorbance was then measured at 410 nm for *p*-nitrophenol with Orlistat employed as the positive control. The percent inhibition and IC_50_ value were calculated by comparing the absorbance to the negative control, which represented 100% enzyme activity, following Eq 3.

$$\text{LI}=\left({\text{A}}_{N}-{\text{A}}_{P}\right)\times 100/{\text{A}}_{\text{N}}$$ (3)

in which A_N_ indicates the absorbance of the negative control in the absence of the peptide, whereas A_P_ indicates the absorbance of activity when the peptide is present.

### Fractionation of lipase inhibitory peptides by ultrafiltration

Ultrafiltration, a membrane-based technique, has been extensively employed in the fractionation of protein hydrolysates to isolate peptides with varying molecular weights. This process is crucial for obtaining bioactive peptides, as their functions are significantly influenced by molecular weight [24, 32]. DLSH peptides were taken from the optimal condition determined by RSM, and four molecular weight cut-off (MWCO) points were used for the fractionation, set at 0.65, 3, 5, and 10 kDa. This allowed five fractions to be isolated as follows: <0.65 kDa, 0.65–3 kDa, 3–5 kDa, 5–10 kDa, and >10 kDa. All fractions were subsequently placed in storage at -20°C until required for experimentation. The protein concentrations of the resulting DLSH fractions were measured along with the IC_50_ values for lipase inhibition. In each of the five fractions, the sample offering the best lipase inhibition underwent further purification in subsequent stages.

### Purification of active fraction by RP-HPLC

Following ultrafiltration, the DLSH fraction (<0.65 kDa), which delivered superior lipase inhibition, underwent further RP-HPLC steps to separate and purify the active components based on their hydrophobicity [33, 34]. It was filtered through a 0.22 μm nylon membrane before undergoing purification *via* RP-HPLC (Spectra System, Thermo Fisher Scientific) employing a C18 column (250 mm × 4.6 mm, Luna 5 μm, Phenomenex, Torrance, CA, USA). Elution gradients of mobile phase A (0.1% (v/v) TFA) and mobile phase B (70% (v/v) acetonitrile in 0.05% (v/v) TFA) were used to separate the peptides when the flow rate was 0.7 mL/min under a 5-phases linear gradient of 100:0% (v/v) A:B subsequently reduced to 88:12% (v/v) A:B at 15 min, then to 75:25% (v/v) A:B at 30 min, prior to 55:45% (v/v) A:B at 40 min, before finally increasing to 100:0% (v/v) A:B at 45 min. A UV detector was used to assess the eluent absorbance at 280 nm, while chromatogram analysis was conducted with ChromQuest software. Finally, the RP-HPLC fractions were gathered and concentrated in order to determine the IC_50_ values for LI.

### Identification of peptides *via* liquid chromatography-quadrupole time-of-flight-tandem mass spectrometry (LC-Q-TOF-MS/MS)

Those peptides fraction obtained *via* RP-HPLC which offered excellent lipase inhibition were then examined for the peptide mass-to-charge ratio (m/z) *via* LC-Q-TOF-MS/MS. This process made use of a Q-TOF mass spectrometer in combination with an electrospray ionization source mass spectrometer (Model Amazon SL, Bruker, Germany). The ESI-Q-TOF mass spectrometer was first calibrated to allow the targeting of peptide chains ranging from 50 m/z to 25,000 m/z, after that the resulting data were interpreted *via de novo* sequencing using the mass differences arising between fragment ion pairs with the aim of determining the residue by establishing the mass of those amino acid residues remaining in the peptide chain. This *de novo* technique allows peptide sequencing to be carried out in the absence of a database containing amino acid sequences. LC-Q-TOF-MS/MS offers several advantages, including high sensitivity and resolution for detecting peptides with low abundance, precise mass measurement to facilitate identification, and the characterization of the primary structure of novel lipase inhibitory peptides [35].

### Peptide synthesis

Starting from the DLSH, it was possible to carry out the chemical synthesis of the lipase inhibitor peptide of interest using the Fmoc solid-phase with an Applied Biosystems Model 433A Synergy peptide synthesizer (Applied Biosystems, Foster City, CA, USA). Analytical mass spectrometry using a quadrupole ion trap Thermo Finnigan™ LXQ™ LC-ESI-MS (San Jose, CA, USA) in combination with a Surveyor HPLC (Thermo Fisher Scientific, San Jose, CA, USA) verified the peptide purification. As a result, it was confirmed that the resulting peptide was of 98.8% purity, in line with the earlier HPLC analysis.

### Bioinformatics tools for the annotation of peptide properties

In order to make modifications to use lipase inhibitory peptides effectively, it is essential to fully understand their properties. It is possible to obtain a profile prediction for any potential lipase inhibitory peptide by checking the online databases. Accordingly, the MS/MS peptide sequences were evaluated in comparison to the results available in the NCBI database using Protein BLAST. The hydrophobicity of peptides can be estimated using Peptide2 (www.peptide2.com), and peptide solubility data can be obtained from the Innovagen server (www.innovagen.com/proteomics-tools), while estimates of the *in silico* peptide toxicity can be obtained from the ToxinPred server (crdd.osdd.net/raghava/toxinpred) using the threshold of the SVM score below zero as indicative of non-toxicity.

### Kinetic study of lipase inhibitory peptides

The inhibitory mechanism of the lipase inhibitor was investigated using the Lineweaver-Burk plot. The preparation procedure of the lipase and substrate solution followed the method outlined in the LI evaluation section. Enzymatic assays involved incubating the lipase with various concentrations of DLSH peptides (0, 0.1, 0.3, and 0.5 mM) and substrate (*p*NPP). The reaction mixtures were incubated under a temperature of 37°C and pH 7.0 for 30 min, while initial velocity rates were determined at different substrate concentrations (0.1, 0.2, 0.3, 0.4, and 0.5 mM). The Lineweaver-Burk plot was constructed by plotting the reciprocal of lipase velocity (1/V) on the y-axis and the reciprocal of substrate concentrations (1/S) on the x-axis. The Michaelis-Menten constant (K_m_) and maximum velocity (V_max_) for the lipase inhibition were determined by analyzing the intercepts on the x-axis and y-axis, respectively. Additionally, the Dixon plot, which illustrates K_m_/V_max_ against peptide concentration, was employed to calculate the inhibition constant (K_i_) value. The x-intercept on the Dixon plot provided a means to assess the binding affinity of the interaction between the lipase and the inhibitor peptide.

### Molecular docking of GRSPDTHSG at the lipase binding site

Molecular docking was performed using AutoDock Vina 1.1.2 [36] to predict the interaction between the GRSPDTHSG peptide and lipase. The 3D crystal structure of the porcine pancreatic lipase-colipase complex (PDB: 1ETH) was obtained from the RCSB Protein Data Bank (https://www.rcsb.org) to serve as the receptor in the docking experiment. The DLSH peptide was constructed with UCSF Chimera 1.17.3, [37] and energy minimization was carried out through multiple steps. The lipase structure was prepared by removing all water molecules and the ligand, followed by the introduction of polar hydrogen atoms and charges using AutoDock Tools 1.5.7. [38]. The position and size of a grid box were designed to cover the pancreatic lipase and colipase domain. The peptide had free access to all possible binding sites, with positions X, Y, and Z at 63.72, 28.47, and 115.76, respectively, and dimensions of X, Y, and Z as 60 Å, 60 Å, and 70 Å, respectively, with a spacing of 1 Å. Docking calculations were performed using AutoDock Vina to rank the binding energy of lipase-peptide interactions, where the lowest binding energy indicates the strongest affinity in the interaction. Subsequently, intermolecular interactions were visualized using ChimeraX 1.5 and Discovery Studio 2021 (BIOVIA Corporate Americas, San Diego, CA, USA) [39].

### Cell culture, differentiation, viability, and lipid inhibition assay

The MTT reduction assay was used to assess the viability of the preadipocyte 3T3-L1 cells (American Type Culture Collection, Manassas, VA, USA). This involved initially culturing the cells in Dulbecco’s modified Eagle’s medium (DMEM) containing 10% fetal bovine serum (FBS) with a density of 5×10^3^ cells per well in a 96-well plate at a temperature of 37°C for 24 h in a 5% carbon dioxide atmosphere prior to treatment with different concentrations of GRSPDTHSG peptide along with simvastatin for 72 h. Following incubation, each well received 10 μL of MTT solution (5 mg/mL), before a further 4 h of incubation at a temperature of 37°C. Then 100 μL of DMSO was added to dissolve the MTT-formazan crystals in the viable cells before measuring the absorbance at 540 nm. The cell viability percentage was then calculated on the basis of the data from the treatment wells for viable cells compared to the results for the control wells.

On the first day, a modified assay proposed by Zhang *et al*. [40] was carried out for the purpose of 3T3-L1 cells cell culture and differentiation, by initially seeding the 3T3-L1 preadipocyte cells in 6-well plates with a density of 1×10^5^ cells per well and then culturing in DMEM with 10% FBS at a temperature of 37°C for 24 h in an atmosphere of 5% carbon dioxide. On the second day, adipocyte differentiation was performed by treating the 3T3-L1 cells with a differentiation medium comprising DMEM with 10% FBS, mixed with 0.5 mM 3-isobutyl-1-methylxanthine (IBMX), 1 μM dexamethasone, and 10 μg/mL insulin. This process took three days, whereupon on day 5 the original medium was removed and a new insulin medium, comprising DMEM with 10% FBS and 10 μg/mL insulin, was used to maintain adipocyte characteristics with a 3-day incubation period. It was then possible to assess the effects of both GRSPDTHSG peptide (0.25, 0.5, 1 mM) and simvastatin (2.5, 5, 10 μM) with testing performed twice in both the differentiation medium and the insulin medium.

Oil Red O staining was then used to evaluate the intracellular lipid accumulation of the mature 3T3-L1 adipocytes. This process required the cells to first be rinsed in PBS and then fixed for 1 h at room temperature with 10% formalin. Once fixed, the cells were then rinsed twice in PBS before staining with 0.35% Oil Red O solution in 60% isopropanol for 1 h at room temperature. The cells were then washed in DI water and examined using a microscope. It was then possible to evaluate the accumulation of lipids by dissolving in isopropanol (100%), and to measure the absorbance at 540 nm. The lipid accumulation was expressed as a percentage in comparison to the control [41].

### Western blot analysis

To determine the protein content in 3T3-L1 differentiated adipocytes, ice-cold NP40 cell lysis buffer containing protease inhibitor cocktail was used to extract samples of 0.25, 0.5, 1 mM of GRSPDTHSG peptide, whereupon the assessment of the protein concentration was carried out *via* absorbance measurement at 280 nm. Once extracted, the proteins of equivalent concentration underwent separation on 12% sodium dodecyl sulfate-polyacrylamide gel electrophoresis (SDS-PAGE) before moving to polyvinylidene fluoride (PVDF) membranes (Millipore, Bedford, MA, USA). These membranes had been blocked at room temperature using 3% skimmed milk in Tris-buffer saline with 0.5% Tween 20 for a period of 2 h. The membranes were then incubated overnight at a temperature of 4°C with the primary antibodies (PPAR-γ, AMPK-α, SREBP-1C and β-actin) diluted at 1:1000. The membranes were then rinsed three times in TBST before incubation for 1 h at room temperature with secondary antibody (horseradish peroxidase (HRP)-conjugated goat anti-rabbit IgG) diluted at 1:5000. An examination of the chemiluminescence properties of the HRP substrate (BIO-HELIX, New Taipei, Taiwan) allowed visualization of the protein bands when using ImageQuant LAS 500 (GE Healthcare, Uppsala, Sweden). The band intensity could then be measured using ImageJ software (version 1.53; National Institutes of Health, MD, USA). The ratio of the target protein intensity to β-actin then enabled the calculation of the relative protein expression level.

### Statistical analysis

The results are shown as mean ± standard error on the basis of experiments performed in triplicate, while one-way ANOVA was employed to evaluate the significance of the differences between sample means. Design-Expert 12 software was used to analyze the response surface methodology while the data were reported and analyzed using SPSS software 22 and GraphPad prism 6. A value of p < 0.05 and p < 0.01 indicated statistical significance.

## Results and discussion

### Protein hydrolysis optimization for DH and LI

#### Preliminary investigations

**Fig 1:**
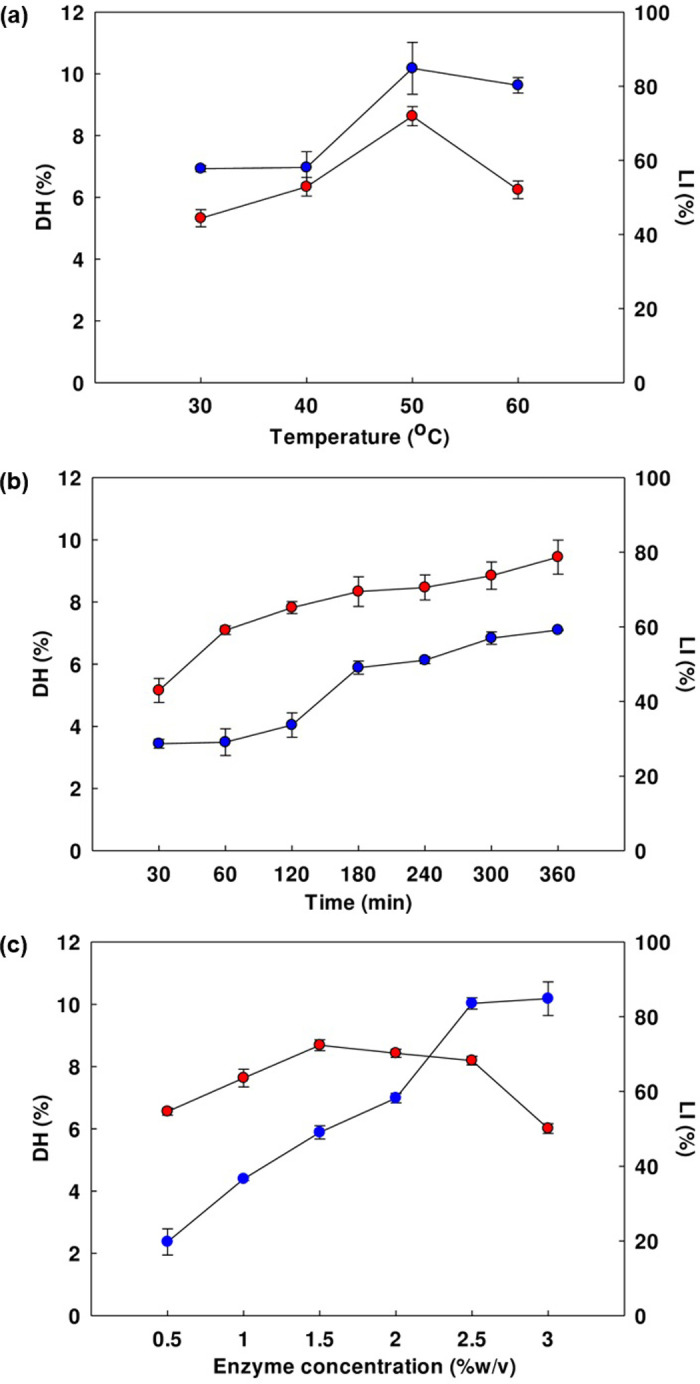
The influence of: (a) temperature, (b) hydrolysis time, and (c) enzyme concentration on the DH (●) and LI (●) of the DLSH. Each data value is presented in the form of mean ± SE and the tests were conducted in triplicate.

Preliminary experimentation was designed to determine the effects of single variables upon the hydrolysis conditions, with the study examining the influence of hydrolysis time, temperature, and enzyme concentration. The central composite design (CCD) experimental design was subsequently based on the findings concerning the interactions of the variables in controlling degree of hydrolysis (DH) and lipase inhibitory activity (LI), as depicted in Fig 1 (S2 Table). The effect of temperature was first determined for the DLSH when the time was 180 min, and the enzyme concentration was maintained at 3% w/v. The degree of hydrolysis represented the quantity of cleaved peptide protein bonds, while lipase inhibition was presented as a percentage. Fig 1(A) depicts for DH and LI increased gradually until the optimal scenario was reached at a temperature of 50°C, whereupon DH was 10.18% and LI was 71.93%. If the temperature was further increased to 60°C, DH and LI began to decline. It could therefore be concluded that slight temperature changes, either rising or falling, reduced the efficiency of trypsin activity in producing lipase inhibitory peptides. Accordingly, 50°C was determined to be the optimal temperature. In Fig 1(B), the effect of hydrolysis time is presented when the temperature is held at 50°C and the enzyme concentration is just 1.5% w/v. As the hydrolysis time was extended, the DH and LI values also rose, reaching a maximum at the time of 360 min. Using palm kernel cake hydrolysates for experimentation, the process of hydrolysis changed over time, with the initial 6 h exhibiting fast hydrolysis, whereupon there was a decline in the rate as the time was extended. The hydrolysis time was therefore set to 6 h, or 360 min [42]. For enzyme concentration analysis, the trials were conducted with a time of 180 min at a temperature of 50°C. It can be seen from Fig 1(C) that as enzyme concentration rose from 0.5% to 3.0% w/v, the DH values also increased, although the LI values were unaffected by this rise in concentration. The maximum value for LI was recorded as 72.38% with the enzyme concentration of 1.5% w/v. Hydrolysis of the protein increased in the presence of additional trypsin, which concurs with earlier reports describing whey protein hydrolysis whereby and increase in the enzyme concentration resulted in greater protease interaction with the protein [43]. To maximize lipase inhibition at minimal cost, the enzyme concentration selected was 1.5% w/v.

#### Response surface methodology (RSM) fitting

RSM enabled the prediction of a model to optimize the production of DLSH peptides. The process used a CCD comprising 20 trial runs in which the three independent variables were temperature (°C, X_1_), time (min, X_2_), and enzyme concentration (% w/v, X_3_). The effects of the independent variables upon DH (Y_1_) and LI (Y_2_) during hydrolysis were examined, and the findings can be observed in Table 1. These results indicated that DH values ranged from 3.64% to 8.18% while LI was in the range of 391.50–893.33 μg/mL. Table 2 presented a summary of the analysis of variance (ANOVA) for DH and LI. The model fitness and the suitability of the independent terms were examined by consideration of the R^2^ coefficient. The regression models were both found to be highly significant (p < 0.0001), while the values for lack of fit values were not significant (p > 0.05), confirming the model fitness to produce predictions for optimal hydrolysis conditions. The R^2^ values indicate that the independent variables and model equations explain 99.09% of the variation in DH, and 99.70% of the variability in LI. Furthermore, the adjusted R^2^ values for both responses matched the R^2^ values. The level of variation unaccounted for by the models remained below 2%, which was indicative of the high degree of accuracy offered by the model. Furthermore, the response variation could be explained to some extent by the statistical significance of the three independent variables in the trials. For DH, significant effects were recorded for the linear terms (X_1_, X_2_, X_3_), the quadratic terms ($X_{1}^{2},X_{2}^{2},X_{3}^{2}$), and the interaction term (X_1_X_2_), whereas there was no significant influence on DH recorded for the remaining interaction terms (X_1_X_3_, X_2_X_3_). In the case of LI, the significant effects were reported for the linear terms (X_1_, X_2_), quadratic terms ($X_{1}^{2},X_{2}^{2},X_{3}^{2}$), and interaction terms (X_1_X_3_, X_2_X_3_), while X_3_ and X_1_X_2_ did not exert any significant influence. The regression analysis demonstrated that by using the second-order polynomial equation, it would be possible to make adjustments to the independent variables which would serve to achieve the aim of maximizing lipase inhibition. The equations for the model could be derived from the coefficients of the linear, quadratic, and interaction terms as indicated below:

$$Y_{1}=-162.52+5.52X_{1}-0.12X_{2}+9.51X_{3}-0.000912X_{1}X_{2}+0.0195{\text{X}}_{1}{\text{X}}_{3}-0.000042{\text{X}}_{2}{\text{X}}_{3}-0.0515{\text{X}}_{1}^{2}-0.00009{\text{X}}_{2}^{2}-2.89{\text{X}}_{3}^{2}$$ (4)

$${\text{Y}}_{2}=19,338.67-502.54{\text{X}}_{1}-24.44{\text{X}}_{2}-2,661.84{\text{X}}_{3}-0.0225{\text{X}}_{1}{\text{X}}_{2}+26.15{\text{X}}_{1}{\text{X}}_{3}+1.54{\text{X}}_{2}{\text{X}}_{3}+4.85{\text{X}}_{1}^{2}+0.0299{\text{X}}_{2}^{2}+262.22{\text{X}}_{3}^{2}$$ (5)

in which Y_1_ indicates the predicted degree of hydrolysis, Y_2_ represents the prediction for LI (IC_50_), and the temperature, time, and enzyme concentration are given respectively by X_1_, X_2_, and X_3_.

**Table 1:** The 20 experimental runs of the independent variables (X_1_, X_2_ and X_3_) and experimental values of responses which included DH (Y_1_) and IC_50_ of LI (Y_2_). Each data values are presented in the form of mean ± SE and the tests were conducted in triplicate.

Run	Type	X_1_ (°C)	X_2_ (min )	X_3_ (%w/v)	Y_1_ (%)	Y_2_ (μg/mL)
1	center	50.00	360.00	1.50	7.95±0.17	410.23±5.05
2		50.00	360.00	1.50	8.18±0.16	391.50±0.84
3		50.00	360.00	1.50	7.78±0.25	399.50±1.86
4		50.00	360.00	1.50	7.70±0.36	398.30±9.51
5		50.00	360.00	1.50	7.59±0.09	406.23±3.96
6		50.00	360.00	1.50	8.06±0.12	424.30±8.36
7	factorial	45.00	300.00	1.00	3.82±0.12	849.10±4.10
8		45.00	300.00	2.00	5.35±0.09	618.63±2.79
9		45.00	420.00	1.00	5.09±0.24	552.20±5.08
10		45.00	420.00	2.00	6.67±0.19	499.83±4.11
11		55.00	300.00	1.00	4.82±0.12	868.70±9.33
12		55.00	300.00	2.00	6.60±0.21	893.33±1.64
13		55.00	420.00	1.00	5.05±0.10	538.33±3.98
14		55.00	420.00	2.00	6.77±0.16	753.97±3.98
15	axial	41.59	360.00	1.50	3.64±0.01	629.87±2.06
16		58.41	360.00	1.50	4.89±0.12	875.40±7.67
17		50.00	259.09	1.50	6.21±0.17	872.20±7.06
18		50.00	460.91	1.50	7.78±0.17	555.60±5.89
19		50.00	360.00	0.66	4.19±0.10	610.17±5.23
20		50.00	360.00	2.34	7.54±0.06	579.63±5.50

**Table 2:** ANOVA for DH (Y_1_) and IC_50_ of LI (Y_2_).

Source	Mean of square		F-value		p-value	
	Y_1_	Y_2_	Y_1_	Y_2_	Y_1_	Y_2_
Model	4.97	7.11×10^4^	120.62	366.40	< 0.0001^*^	< 0.0001^*^
X_1_	1.43	6.57×10^4^	34.60	338.80	0.0002^*^	< 0.0001^*^
X_2_	2.32	1.47×10^5^	56.35	758.70	< 0.0001^*^	< 0.0001^*^
X_3_	10.98	646.06	266.46	3.33	< 0.0001^*^	0.0980
X_1_X_2_	0.599	364.91	14.55	1.88	0.0034^*^	0.2002
X_1_X_3_	0.019	3.42×10^4^	0.46	176.29	0.5123	< 0.0001^*^
X_2_X_3_	0.00	1.70×10^4^	0.0003	87.77	0.9864	< 0.0001^*^
$X_{1}^{2}$	23.93	2.12×10^5^	580.90	1093.25	< 0.0001^*^	< 0.0001^*^
$X_{2}^{2}$	1.51	1.67×10^5^	36.59	860.36	0.0001^*^	< 0.0001^*^
$X_{3}^{2}$	7.53	6.19×10^4^	182.83	319.18	< 0.0001^*^	< 0.0001^*^
Residual	0.041	194.03				
Lack of fit	0.032	256.30	0.623	1.95	0.6915	0.2414
Pure error	0.051	131.75				
Cor total	2.37	33.78×10^4^				
Std. Dev.	0.20	13.93				
R^2^	0.9909	0.9970				
Adj. R^2^	0.9827	0.9943				

^*^ variables found to exert a significant effect on the response (p <0.05)

RSM was constructed to efficiently predict the optimal condition for producing bioactive peptides, thus reducing the number of experiments required and providing insights into the complex interactions between variables. Studies by Bukhaiti *et al*. [19] and Ding *et al*. [44] have demonstrated the efficacy of RSM in predicting optimal conditions for protein hydrolysate preparation, resulting in antioxidant and diabetic inhibitory activities, respectively. Additionally, the finding of salmon bone protein [45] exhibited how RSM can adequately predict the optimal conditions for producing bioactive peptides with antihypertension contributing to the enhancement of the bioactivity of protein hydrolysate against diseases.

#### Response surface and contour plots

**Fig 2:**
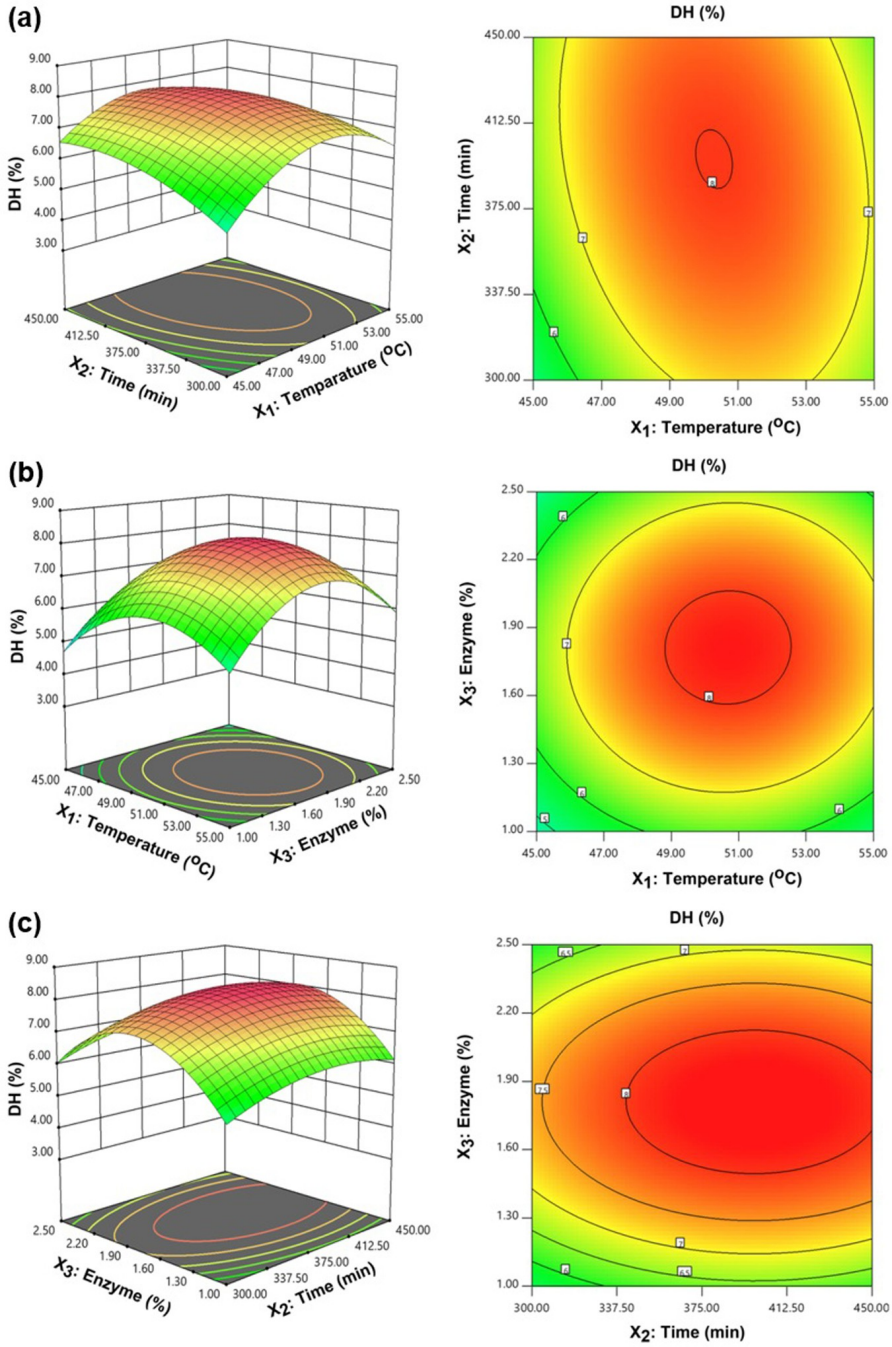
3D response surface plots and 2D contour plots for DH with the various factors: (a) The combined influence of temperature and time; (b) The combined influence of temperature and enzyme concentration, and (c) The combined influence enzyme concentration and time.

**Fig 3:**
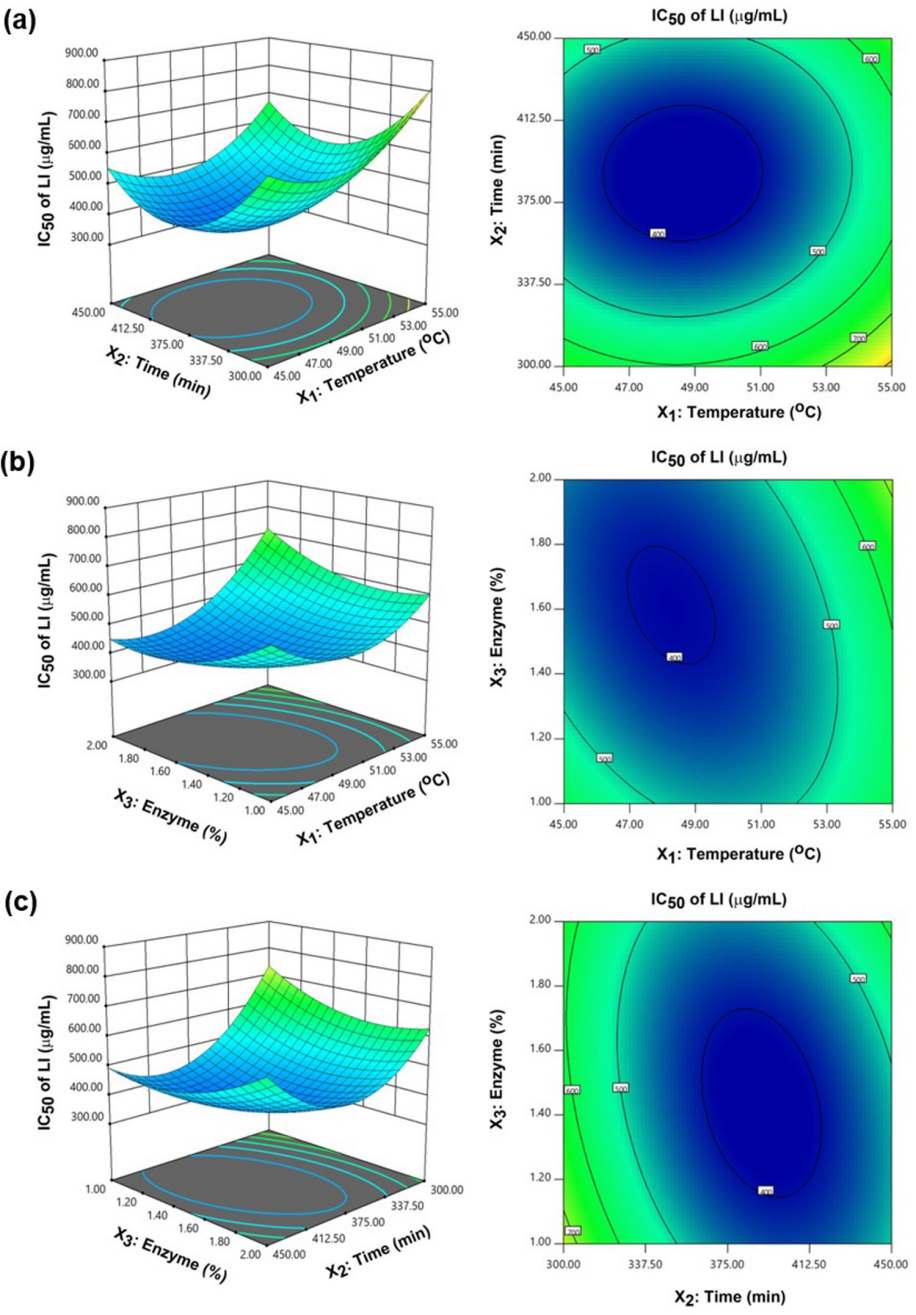
3D response surface plots and 2D contour plots for LI with the various factors: (a) The combined influence of temperature and time; (b) The combined influence of temperature and enzyme concentration, and (c) The combined influence of time and enzyme concentration.

3D response surface plots and 2D contour plots were used to visualize the relationships or interactions among variables. Construction of the 3D plots was based on Eqs 4 and 5, in which the LI or DH values were plotted on the z-axis, fixing one variable, and allowing the remaining two to vary along the x- and y-axes. Meanwhile, the 2D plots were constructed on the basis of the model equations in which the values for LI and DH are placed on a curved radius. Fig 2A–2C presents the influence of the interaction between time and temperature on DH. It can be seen that DH would initially increase with temperature before subsequently decreasing, whereas with time, DH increased steadily. The quadratic effect on DH of temperature and enzyme concentration was also investigated, revealing that DH increased with time and enzyme concentration but the effect of higher enzyme concentrations was to decrease DH while the effect of a longer time was to maintain DH at a steady value. A quadratic effect with all three factors was seen for the IC_50_ of LI. Those IC_50_ values showed a tendency to decline until the various values of the factors reached their optimal point, whereupon IC_50_ increased as the factor values also rose, as shown in Fig 3A–3C.

#### Validation test

Design-Expert was employed to optimize the conditions for hydrolysis when the defined aim was to maximize DH and achieve the minimum IC_50_ for LI. The optimal conditions to maximize DH included enzyme concentration of 1.81% w/v, temperature of 50.30°C, and hydrolysis time of 389.27 min. The predicted DH (8.29%) almost matched the experimental DH (8.20%). To minimize IC_50_ for LI, a value of 375 μg/mL was predicted when the enzyme concentration was 1.61% w/v, the temperature was 49.03°C, and the hydrolysis time was 387.06 min. In the experiment under optimal conditions, the LI achieved was a little lower at 368.07 μg/mL. This was significantly different from the scenario which optimized DH, since that case resulted in lipase inhibitory peptides producing a higher IC_50_ value of 413.23 μg/mL. It can be seen that while the hydrolysis reaction does generate bioactive peptides, there is no relationship between the DH values and the lipase inhibition. With incremental increases in hydrolysis time, the lipase inhibitory peptide obtained from camel milk lowered its inhibition during trypsin hydrolysis [46]. Furthermore, longer hydrolysis times might have a damaging effect on the bioactive peptides [47]. Gao *et al*. [48] found that longer hydrolysis times could lead to greater DH, but RSM optimization results did not find that this was associated with improved angiotensin-converting enzyme (ACE) inhibitory activity, but instead that the amino acid composition, peptide size, or binding sites may have a much greater influence on the biological activity observed [49].

### Fractionation of lipase inhibitory peptides by ultrafiltration

Preparation of the DLSH bioactive peptides was carried out using the optimal conditions for LI. The peptides then underwent fractionation to obtain five fractions separated by molecular weight cut-off boundaries: >10 kDa, 5–10 kDa, 3–5 kDa, 0.65–3 kDa, and <0.65 kDa. Each of the ultrafiltered fractions exhibited the capacity for lipase inhibition, but the smallest size fraction (<0.65 kDa), delivered the strongest lipase inhibition, achieving an IC_50_ value of 4.42 μg/mL, as shown in Table 3 (S3 Table). This may be attributable to the increased ability of smaller peptides to access the lipase binding sites. It was apparent that smaller peptides offer superior performance in terms of ACE inhibition and antioxidant activity [28, 50].

**Table 3:** LI (IC_50_) results of DLSH ultrafiltered fractions.

Molecular weight (kDa)	Protein content (mg)	IC_50_ of LI (μg/mL)
DLSH hydrolysate	1,969.30 ± 58.94	368.07 ± 3.69
>10	568.38 ± 5.78	263.10 ± 5.01^a^
5–10	398.07 ± 13.23	86.87 ± 1.33^b^
3–5	462.01 ± 8.76	58.02 ± 0.82^c^
0.65–3	158.42 ± 4.55	24.83 ± 0.41^d^
<0.65	59.43 ± 2.89	4.42 ± 0.06^e^

All values are presented in the form of LI mean (IC_50_ value) ± SE with the tests performed in triplicate. When a letter in superscript is presented in the same column, this is indicative of a significant difference when applying Duncan’s multiple range test (p < 0.05).

### Purification of active fraction by RP-HPLC

**Fig 4:**
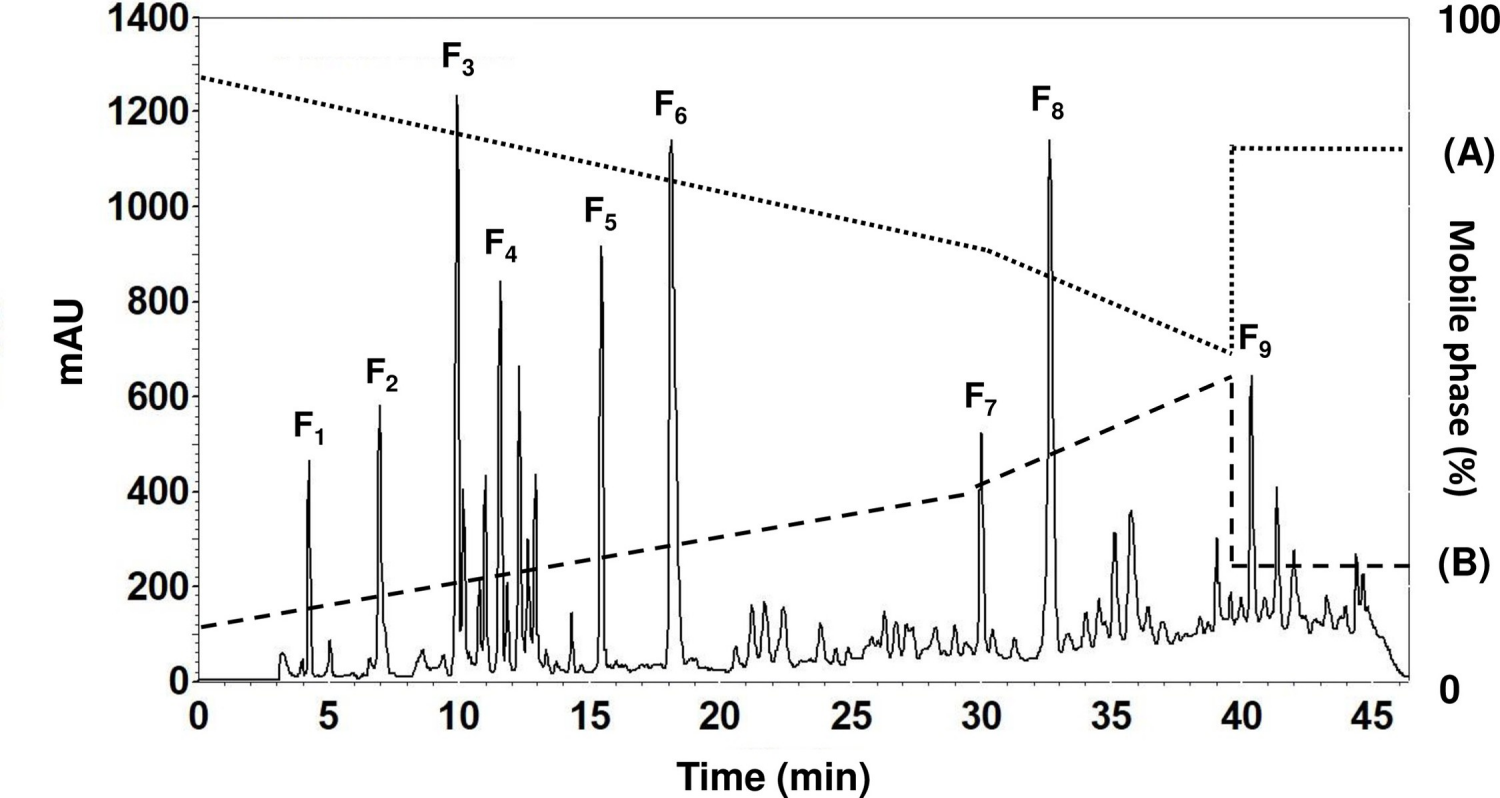
The RP-HPLC chromatogram for fraction <0.65 kDa of the original DLSH ultrafiltered fraction.

Using the polarity property, the smallest ultrafiltered fraction, <0.65 kDa, underwent further separation *via* gradient elution through RP-HPLC. Hydrophilic molecules were indicated by low retention times, while longer retention times were associated with hydrophobic molecules. The gradient elution process was carried out over a period of 45 min, whereupon the resulting RP-HPLC chromatogram profiles can be seen in Fig 4 depicting nine principal fractions, designated as F_1_ to F_9_. These fractions were collected and their lipase inhibition was assessed at equal concentrations of 1 μg/mL of peptides. The outcomes are shown in Table 4 (S4 Table). The short retention time for the elution of the fraction F_1_ confirms the presence of hydrophilic or polar amino acid residues. This fraction showed the greatest LI at 64.31 ± 1.16% while the IC_50_ value was found to be 0.193 ± 0.013 μg/mL. This differed from Mudgil *et al*. [47] and Ngoh *et al*. [51] who claimed that in their studies involving peptides derived from camel milk and pinto bean hydrolysates, the strongest lipase inhibition activity occurred in fractions exhibiting high levels of hydrophobic, rather than hydrophilic, amino acid residues. Considering a broader range of studies, however, a majority are unable to confirm that hydrophobic molecules have a positive influence on lipase inhibition. In the emulsified condition, a lipase typically acts at the interface between the lipid and the water. Therefore, it is possible for hydrophilic molecules to interact with the amphipathic structure of the lipase, thus reducing the lipase activity. Accordingly, the HPLC fraction F_1_ was chosen for further analysis to determine the identity of the amino acid sequences *via* LC-Q-TOF-MS/MS.

**Table 4:** LI results of RP-HPLC fraction of DLSH.

Fraction	Retention time (min)	Protein content (mg)	LI (%)
F_1_	4.05–4.44	1.90 ± 0.88	64.31 ± 1.16^a^
F_2_	6.65–7.33	2.50 ± 0.33	51.15 ± 1.74^b^
F_3_	9.53–10.31	5.38 ± 0.99	33.00 ± 1.22^c^
F_4_	11.22–11.90	4.06 ± 0.67	34.73 ± 1.16^cd^
F_5_	15.12–15.80	5.57 ± 1.77	41.42 ± 1.32^e^
F_6_	17.63–18.59	10.34 ± 3.62	37.55 ± 0.43^cde^
F_7_	27.21–32.85	3.08 ± 0.78	38.44 ± 0.59^de^
F_8_	33.27–34.51	9.23 ± 0.90	34.11 ± 0.69^cd^
F_9_	40.14–40.59	2.79 ± 0.33	27.28 ± 2.66^f^

The inhibitions were measured at the same concentration at 1 μg/mL of peptide. The results are presented in the form of mean ± SE (n = 3) and the superscripts a-f on means represent significant difference (p < 0.05).

### Identification of peptide sequences and bioinformatic predictions of peptide properties

**Fig 5:**
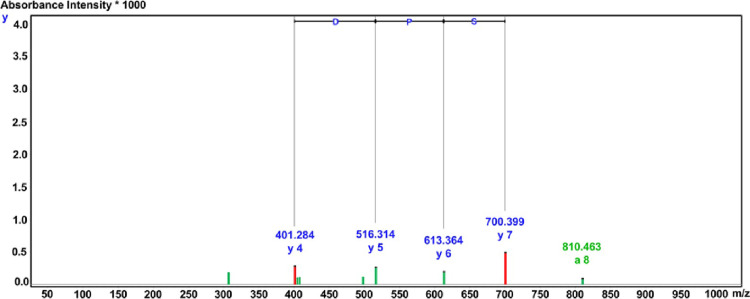
Amino acid sequence mass fragmentation spectrum and analysis for HPLC fraction F_1_ (GRSPDTHSG) by LC-Q-TOF-MS/MS.

For the amino acid sequence identification, analysis of the lipase inhibitory peptide of the HPLC fraction F_1_ was carried out *via* LC-Q-TOF-MS/MS de novo sequencing on the basis of the *Ocimum* genus. Fig 5 presents the mass spectral identification given as Gly-Arg-Ser-Pro-Asp-Thr-His-Ser-Gly (GRSPDTHSG), with the peptide determined to comprise nine amino acids at a molecular weight of 912.91 Da. The LI of the synthetic GRSPDTHSG peptide was assessed, with the IC_50_ value measuring 0.255 ± 0.007 mM. The location was aligned in the homologous region by Protein BLAST. This GRSPDTHSG peptide exhibited 85.71% similarity to the RNA polymerase β-subunit protein derived from *Occimum* spp., while also showing 85.71% similarity to the lectin 2 protein from *Occimum basilicum* (S5 Table). The full properties and stabilities of the peptides required for the preparation of functional peptides can be determined *via* bioinformatic searching. For instance, the data obtained from the Innovagen server are presented in Table 5 and show that the GRSPDTHSG peptide offers good solubility in water, although this decreased at concentrations exceeding 10 mg/mL.

**Table 5:** The GRSPDTHSG peptide properties profiles.

List of properties	Peptide properties
Peptide	GRSPDTHSG
Solubility^a^	Good water solubility
Hydrophobicity (%)^b^	11.11%
Toxicity (SVM score)^c^	Non-Toxic (-0.46)

^a^Data obtained from the Innovagen server concerning peptide solubility.

^b^Calculations were performed on the peptide property calculator (www.peptide2.com).

^c^Outcomes from peptide toxicity analysis was performed using the ToxinPred server

The vital consideration in developing bioactive peptides for the functional food sector is toxicity. The creation of *in silico* models allows the ToxinPred server to be used to predict peptide toxicity. In this research, the peptides were derived from a plant source, while the protease used in preparing the protein hydrolysate was derived from an animal source. Both sources have been widely used in the food sector and present no immediate health concerns. The SVM scores were below the zero level for the thresholds (SVM = -0.46; Table 5), and therefore the prediction indicated non-toxicity. The peptide should therefore be suitable for food applications, although further investigation is still required through the use of *in vitro* assays and *in vivo* modeling prior to actual human consumption.

### Inhibition kinetics of lipase inhibitory peptides

**Fig 6:**
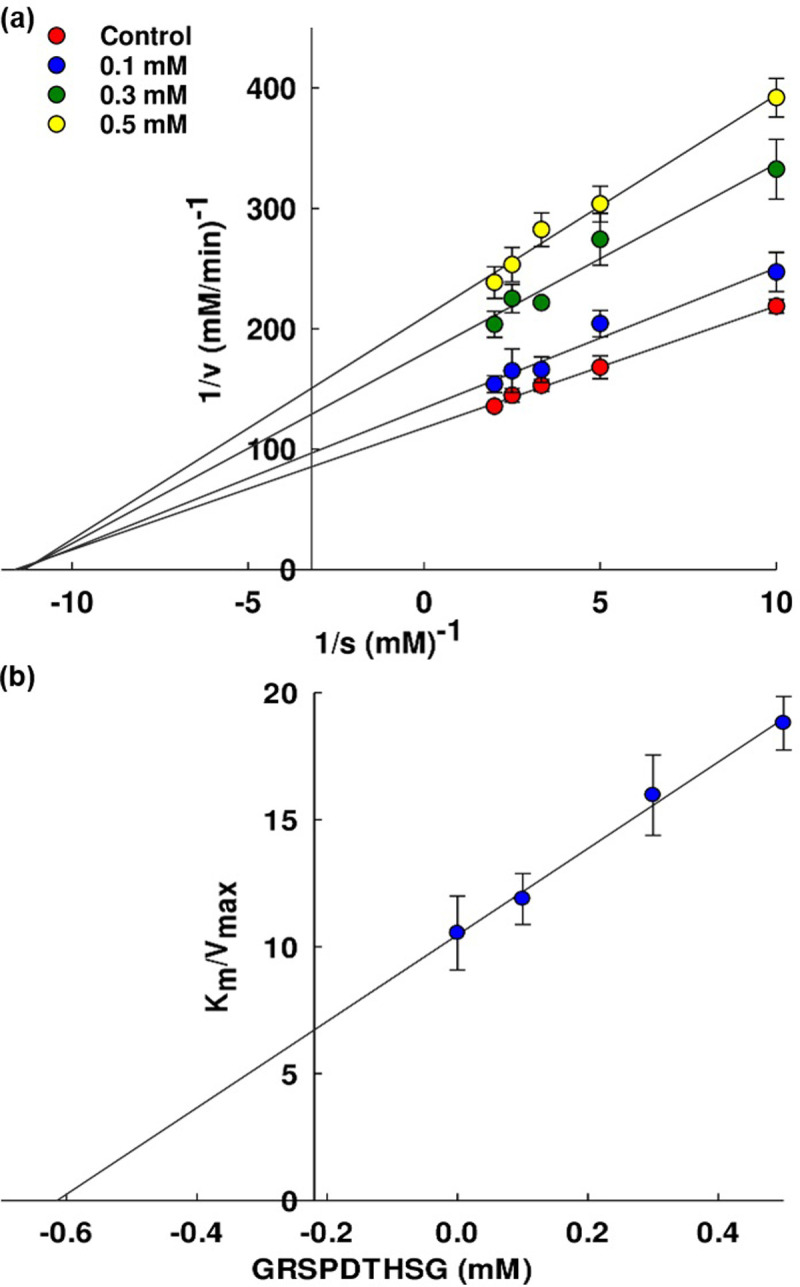
(a) The Lineweaver-Burk plot showing lipase inhibition with the GRSPDTHSG peptide both present and absent. 1/V and 1/S are the respective reciprocals of the velocity and substrate. Each of the points is presented in the form of mean value ± SE. Deviation with tests performed in triplicate, and (b) the Dixon plots showed the determination of the inhibitor constant (K_i_) in the case of the non-competitive inhibition by GRSPDTHSG.

The Lineweaver-Burk plot was used to evaluated the inhibition mode of the GRSPDTHSG peptide obtained from DLSH, and the outcome can be observed in Fig 6(A) (S6 Table). The plot of the inhibition activity was created by altering the concentration of the substrate while maintaining the peptide concentration at the same level throughout (0, 0.1, 0.3 and 0.5 mM). The resulting plot was indicative of non-competitive inhibition involving binding at the non-active site of the free lipase and also to the lipase-substrate complex. The peptide accordingly acted as a lipase inhibitor by taking the form of enzyme-inhibitor (EI) complexes and enzyme-substrate-inhibitor (ESI) complexes which served to restrict the process of catalysis when the reaction takes place. The kinetic parameters of the lipase activity are presented in Table 6. The K_m_ value was consistently recorded at 89.70 ± 6.67 μM, while the V_max_ for lipase activity was measured as 8.51 ± 0.55 μM/min. In the presence of increasing peptide concentrations, there was a decrease in reaction velocity. For varying peptide concentrations of 0.1, 0.3, and 0.5 mM, the V_max_ values dropped to 7.55 ± 0.46, 5.62 ± 0.31, and 4.77 ± 0.01 μM/min, respectively, indicating a non-competitive mode of inhibition. The inhibition constant (K_i_), which represents the binding strength between the lipase and the inhibitor peptide, was determined to be 0.61 ± 0.09 mM, as shown in Fig 6(B) (S6 Table). A low K_i_ value suggests a tight bond and strong inhibitory activity. However, there has been little previous research into the lipase inhibition mode of bioactive peptides, although non-competitive inhibition was reported in the case of fish protein hydrolysate [52] and tripeptide glutathione [53]. In contrast, competitive inhibition of lipase was observed in the case of the phenolic compounds found in muscadine grapes, [54] while non-competitive inhibition was seen in *Camellia nitidissima* chi flower extract, [55] almond cake skim [56].

**Table 6:** Kinetic parameters of lipase activity at varying GRSPDTHSG peptide concentrations.

Kinetic parameter	Control	GRSPDTHSG (mM)		
		0.1	0.3	0.5
K_m_ (μM)	89.70 ± 6.67	89.70 ± 6.67	89.70 ± 6.67	89.70 ± 6.67
V_max_ (μM/min)	8.51 ± 0.55	7.55 ± 0.46	5.62 ± 0.31	4.77 ± 0.01
K_i_ (mM)	-	0.61 ± 0.09	0.61 ± 0.09	0.61 ± 0.09

The results are findings are expressed in the form of mean ± SE (n = *3)*.

### Molecular docking of the GRSPDTHSG peptide with lipase

**Fig 7:**
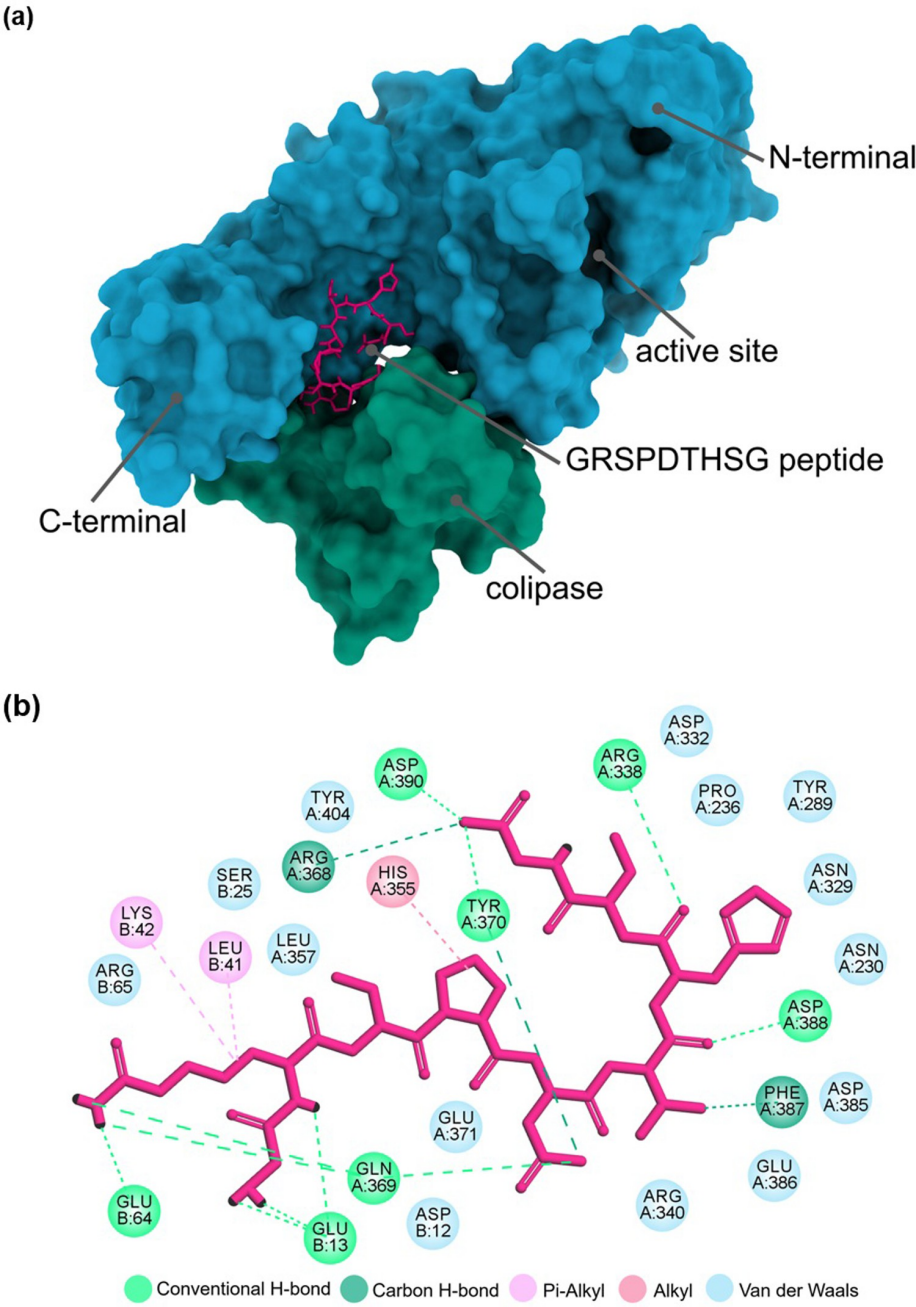
Molecular interaction between the GRSPDTHSG peptide and the porcine pancreatic lipase-colipase complex (1ETH). (a) Broad perspective for the 3D illustration of the interactions between peptide-lipase complex. (b) 2D diagram of the anticipating interaction.

Until now, there has been limited exploration into the intermolecular mechanism governing the interaction between lipase and lipase inhibitory peptides derived from food proteins. Understanding these interactions is crucial for designing more effective peptide inhibitors. Using AutoDock Vina to determine binding affinity scores, the GRSPDTHSG peptide emerged as a prospective inhibitor of lipase activity, with a calculated binding energy of -7.1 kcal/mol. Fig 7, illustrating optimal binding conformations and sites, was generated using the Discovery Studio program. Analysis of lipase-peptide interactions revealed no binding to the catalytic sites (Ser153, Asp177, and His264) of porcine pancreatic lipase [57]. This implies that the GRSPDTHSG peptide does not compete with the lipase-substrate at the same active site, a deduction supported by the Lineweaver-Burk plot in the kinetic results, indicating a noncompetitive mechanism.

The interaction between the GRSPDTHSG peptide and lipase residues primarily involved hydrogen bonding, followed by hydrophobic interactions and van der Waals forces. Among these interactions, the hydrogen bond seems to significantly contribute to lipase inhibitory activity. The peptide formed sixteen hydrogen bonds with the amino acid residues of lipase, along with three hydrophobic interactions, detailed in Table 7. Importantly, the peptide exhibited binding to two distinct sites on the lipase structure. At the C-terminal site (domain A), it established hydrogen bonds with Asp338, Arg368, Gln369, Tyr370, Phe387, Asp388, and Asp390 residues, coupled with a hydrophobic interaction with His355. Simultaneously, at the colipase binding site (domain B), it formed hydrogen bonds with Glu13 and Glu64, along with hydrophobic interactions with Leu41 and Lys42. Van der Waals forces facilitated interactions around the peptide molecule, contributing to a robust binding involving the 14 residues.

**Table 7:** Intermolecular interactions between the GRSPDTHSG peptide and lipase residues.

Peptide	Lipase (domain)	Interacting type	From chemistry	To chemistry	Distance (Å)
Gly1	Glu13 (B)	Conventional hydrogen bond	A:Gly1:H2 (H-donor)	B:Glu13:OE2 (H-acceptor)	2.28910
	Glu13 (B)	Conventional hydrogen bond	A:Gly1:H3 (H-donor)	B:Glu13:OE2 (H-acceptor)	2.46268
Arg2	Gln369 (A)	Conventional hydrogen bond	A:Arg2:HH21 (H-donor)	A:Gln369:O (H-acceptor)	2.88199
	Gln369 (A)	Conventional hydrogen bond	A:Arg2:HH22 (H-donor)	A:Gln369:O (H-acceptor)	2.70509
	Glu13 (B)	Conventional hydrogen bond	A:Arg2:H (H-donor)	B:Glu13:OE2 (H-acceptor)	3.03411
	Leu41 (B)	Hydrophobic (Alkyl)	A:Arg2 (Alkyl)	B:Leu41 (Alkyl)	5.35261
	Lys42 (B)	Hydrophobic (Alkyl)	A:Arg2 (Alkyl)	B:Lys42 (Alkyl)	4.89542
	Glu64 (B)	Conventional hydrogen bond	A:Arg2:HH22 (H-donor)	B:Glu64:OE1 (H-acceptor)	2.61393
Pro4	His355 (A)	Hydrophobic (Pi-Alkyl)	A:His355 (Pi-Orbitals)	A:Pro4 (Alkyl)	5.12589
Asp5	Gln369 (A)	Conventional hydrogen bond	A:Asp5:OD2 (H-donor)	A:Gln369:O (H-acceptor)	3.05249
	Tyr370 (A)	Carbon hydrogen bond	A:Tyr370:CA (H-donor)	A:Asp5:OD2 (H-acceptor)	3.59018
Thr6	Phe387 (A)	Carbon hydrogen bond	A:Phe387:CA (H-donor)	A:Thr6:OG1 (H-acceptor)	3.45350
	Asp388 (A)	Conventional hydrogen bond	A:Asp388:HN (H-donor)	A:Thr6:O (H-acceptor)	2.07039
	Asp388 (A)	Conventional hydrogen bond	A:Asp388:OD2 (H-donor)	A:Thr6:O (H-acceptor)	3.18745
His7	Asp338 (A)	Conventional hydrogen bond	A:Arg338:HH11 (H-donor)	A:His7:O (H-acceptor)	2.94354
	Asp338 (A)	Conventional hydrogen bond	A:Arg338:HH12 (H-donor)	A:His7:O (H-acceptor)	3.08197
Gly9	Arg368 (A)	Carbon hydrogen bond	A:Arg368:CD (H-donor)	A:Gly9:OXT (H-acceptor)	3.29523
	Tyr370 (A)	Conventional hydrogen bond	A:Gly9:OXT (H-donor)	A:Tyr370:OH (H-acceptor)	3.15658
	Asp390 (A)	Conventional hydrogen bond	A:Asp390:OD2 (H-donor)	A:Gly9:OXT (H-acceptor)	3.18376

Considering the binding interaction, it is evident that the peptide disrupts the stability of the lipase-colipase complex, facilitating efficient activity through a noncompetitive mode at both the C-terminal and colipase sites. Previous investigations on the canary seed peptide [58], and gallotannins [59] have provided in-depth insights into the binding mechanism, yielding consistent results. These studies have further substantiated the occupancy of binding sites within the pancreatic lipase and colipase domains. Colipase acts as a cofactor for pancreatic lipase, inducing an active conformational alteration in the lipase-colipase complex upon association with the lipid-water interface [60]. The preceding molecular docking analysis has illuminated an escalated interaction between arginine and residues crucial for maintaining pancreatic lipase activity, implying that peptides rich in arginine could potentially serve as inhibitors of pancreatic lipase [61]. In the case of the DLSH peptide, the Arg2 residue engaged in hydrophobic interactions with Leu41 and Lys42 of the lipase, concurrently forming hydrogen bonds with the charged residues (Glu13 and Glu64). Moreover, arginine-containing peptides derived from seabuckthorn seed meal seem to play a significant role as inhibitors, demonstrating robust hydrophobic interactions with hydrophobic residues and establishing hydrogen bonds with charged residues in the lipase [62]. The formation of hydrogen bonds with the colipase residue Glu64, a hydrophilic surface critical for the interaction of colipase with the C-terminal site, contributes significantly to the lipolysis reaction [63].

### The influence of the GRSPDTHSG peptide on lipid accumulation in 3T3-L1 cells

**Fig 8:**
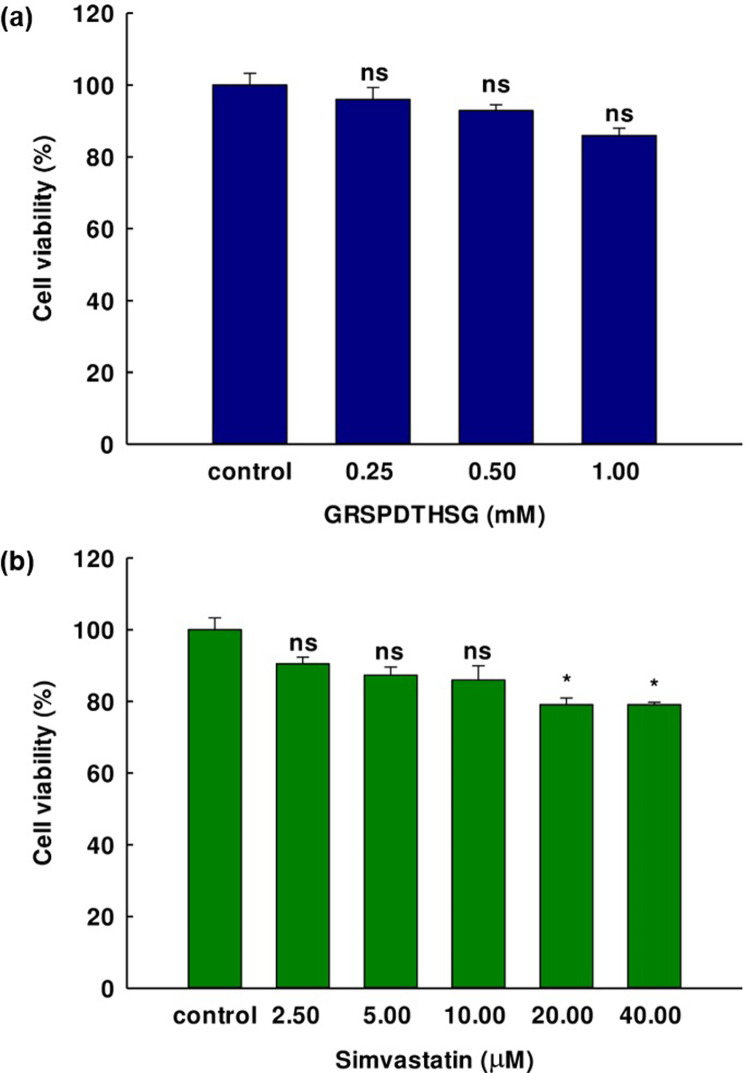
3T3-L1 cell viability after treatment for 72 h with different concentrations of (a) GRSPDTHSG, and (b) simvastatin. The graph presents the mean ± SE (n = 3). “ns” indicates not significance, while “*” indicates a significant difference compared to the untreated control (p < 0.001).

**Fig 9:**
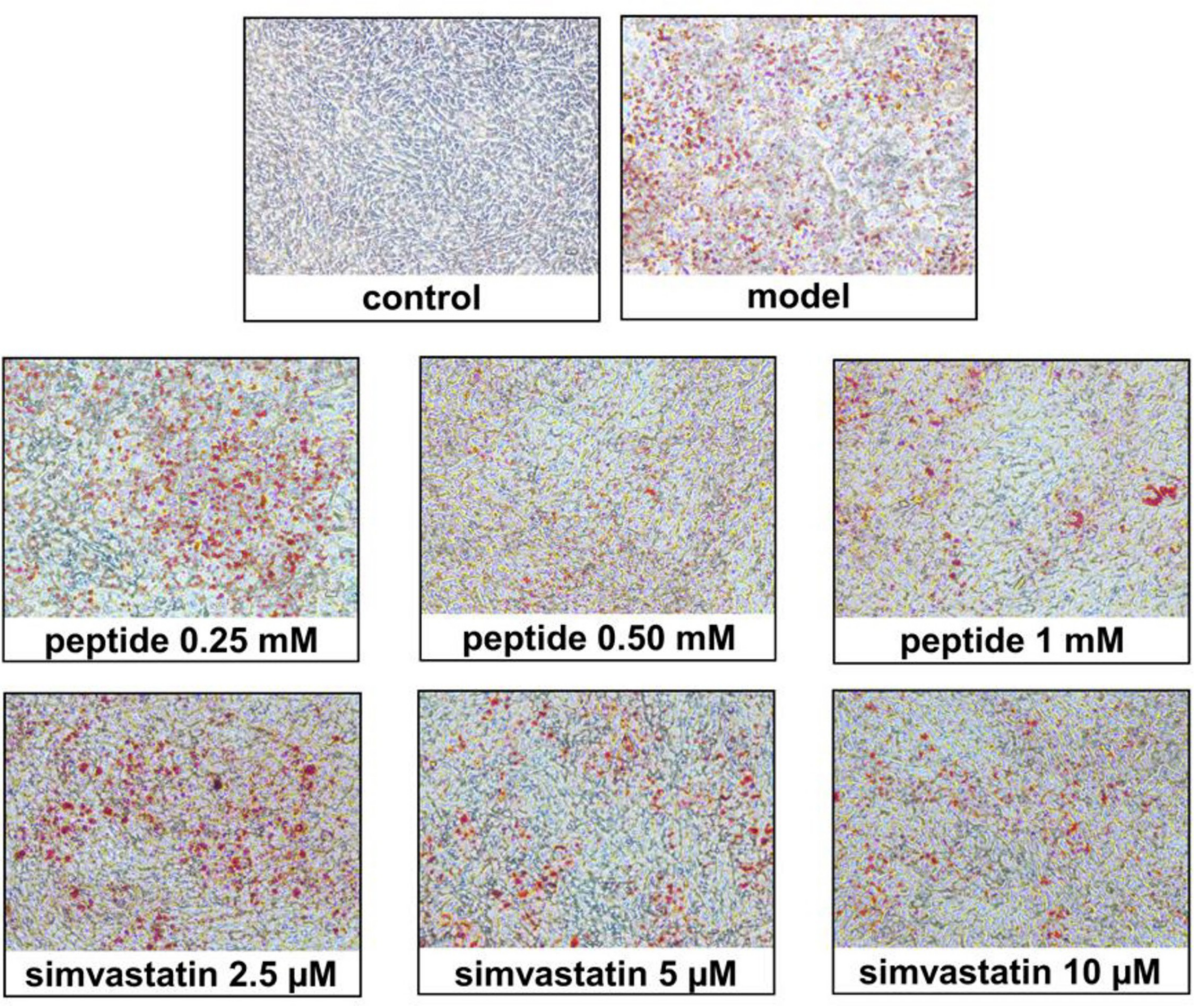
Microscopic image of 3T3-L1 cells stained with Oil Red O (at 200 × magnification).

The MTT assay was performed using 3T3-L1 cells to assess the cytotoxic effects of the GRSPDTHSG peptide and simvastatin. The findings in Fig 8A and 8B (S7 Table) indicate that the GRSPDTHSG peptide had no significant effect on the viability of 3T3-L1 cells at concentrations below 1 mM, compared to the untreated control. As for Simvastatin, it demonstrated no toxicity until a concentration of 10 μM was reached. Adipogenesis inhibition evaluation for the GRSPDTHSG peptide and simvastatin involved treating 3T3-L1 preadipocytes with different GRSPDTHSG peptide concentrations (0.25, 0.5, 1 mM) and simvastatin concentrations (2.5, 5, 10 μM) with the differentiation medium (day 2) and the insulin medium (day 5). The 3T3-L1 cells were first of all induced into adipocyte cells in the form of intracellular lipid droplets, prior to examination *via* Oil Red O staining. Under microscopy, it could be seen that increasing the GRSPDTHSG peptide or simvastatin concentration caused a decline in the liquid droplet quantity, as shown in Fig 9 (S8 Table). In the undifferentiated control, the oil droplets were barely visible at 36.08 ± 4.24%, while in contrast, the lipid droplets in the control model were clearly apparent as differentiated cells. However, one effect of the GRSPDTHSG peptide was significantly lower lipid accumulation as the concentration of the peptide increased. Strong inhibition of 3T3-L1 adipocyte cell differentiation was observed for the highest peptide concentration at 1 mM. However, there was also a decline in intracellular lipids to just 46.83 ± 4.01% when compared to the control, while a reduction in lipid accumulation to 51.66 ± 1.81% was seen for 10 μM of simvastatin. Accordingly, it would seem that the GRSPDTHSG peptide might limit 3T3-L1 preadipocyte differentiation, thereby restricting the accumulation of intracellular lipids.

Although we believe there have been no previous studies detailing the GRSPDTHSG peptide, it can be considered as a suitable novel peptide capable of addressing the problem of obesity. The literature does contain references to peptides which act in a similar manner to affect preadipocytes during their initial proliferation. These include black soybean-derived IQN which has been shown to inhibit 3T3-L1 preadipocytes in mice [64], marine-derived VAP, AP, and AKK which have been shown to exhibit inhibitory effects with human white pre-adipocyte [65]. It was noted by Zhang *et al*. [40] that the synthetic decapeptide, LLVVYPWTQR, derived from *C*. *pyenoidose* at 600 μg/mL, was able to reduce the intracellular lipid accumulation to 27.91% in 3T3-L1 adipocytes. Meanwhile, Kim *et al*. [66] reported that a peptide derived from extract of boiled tuna (DIVDKIEI) was effective in inhibiting 3T3-L1 preadipocyte differentiation while lowering both triglyceride levels and the uptake of glucose. In addition, a strong inhibitory influence on 3T3-L1 preadipocyte proliferation was shown by four novel peptides derived *via* pepsin digestion hydrolysis from the *Spirulina platensis* protein, with two peptides, NPVWKRK and CANPHELPNK, playing key roles and potentially limiting triglyceride accumulation [67]. Kim *et al*. [68] was also able to create a novel synthetic hexapeptide, GAGVGY, which was obtained from the repetitive amino acid sequences in fibroin. GAGVGY was shown to enhance the transport of glucose and support the lipid metabolism in 3T3-L1 adipocytes. While little is known about the precise structure activity relationships of peptides offering anti-obesity properties, it appears from the evidence to date that such peptides contain hydrophobic and alkaline amino acid residues. In particular, the most prevalent among the hydrophobic amino acid residues in the peptides were Pro, Val, Ile, and Leu, while reported alkaline amino acid residues included Lys and His [69, 70]. Anti-obesity activity is known to be supported by the presence in peptides of hydrophobic amino acid residues. This is because hydrophobic peptides are more readily able to penetrate cell membranes in addition to improving lipid solubility [71–73]. While the amino acid sequence of DLSH peptide (GRSPDTHSG) does contain a mixture of hydrophilic and polar amino acids, it is essential to consider the natural environment in which pancreatic lipase primarily operates. Pancreatic lipase functions within the polar environment of the small intestine, where dietary fats (triglycerides) are emulsified in an aqueous setting with the assistance of bile salts. This emulsification results in the formation of small fat droplets within this watery environment. Consequently, the hydrophilic properties of peptide sequences are of paramount importance, as they facilitate interactions within this aqueous environment and are integral to the digestive processes mediated by pancreatic lipase. It is still necessary, however, to examine further the structure activity relationship for peptides which might be employed to treat obesity, since the peptide sequence and protein source may also play significant roles in addition to the amino acid composition.

### Effects of the GRSPDTHSG peptide on protein expression in 3T3-L1 cells

**Fig 10:**
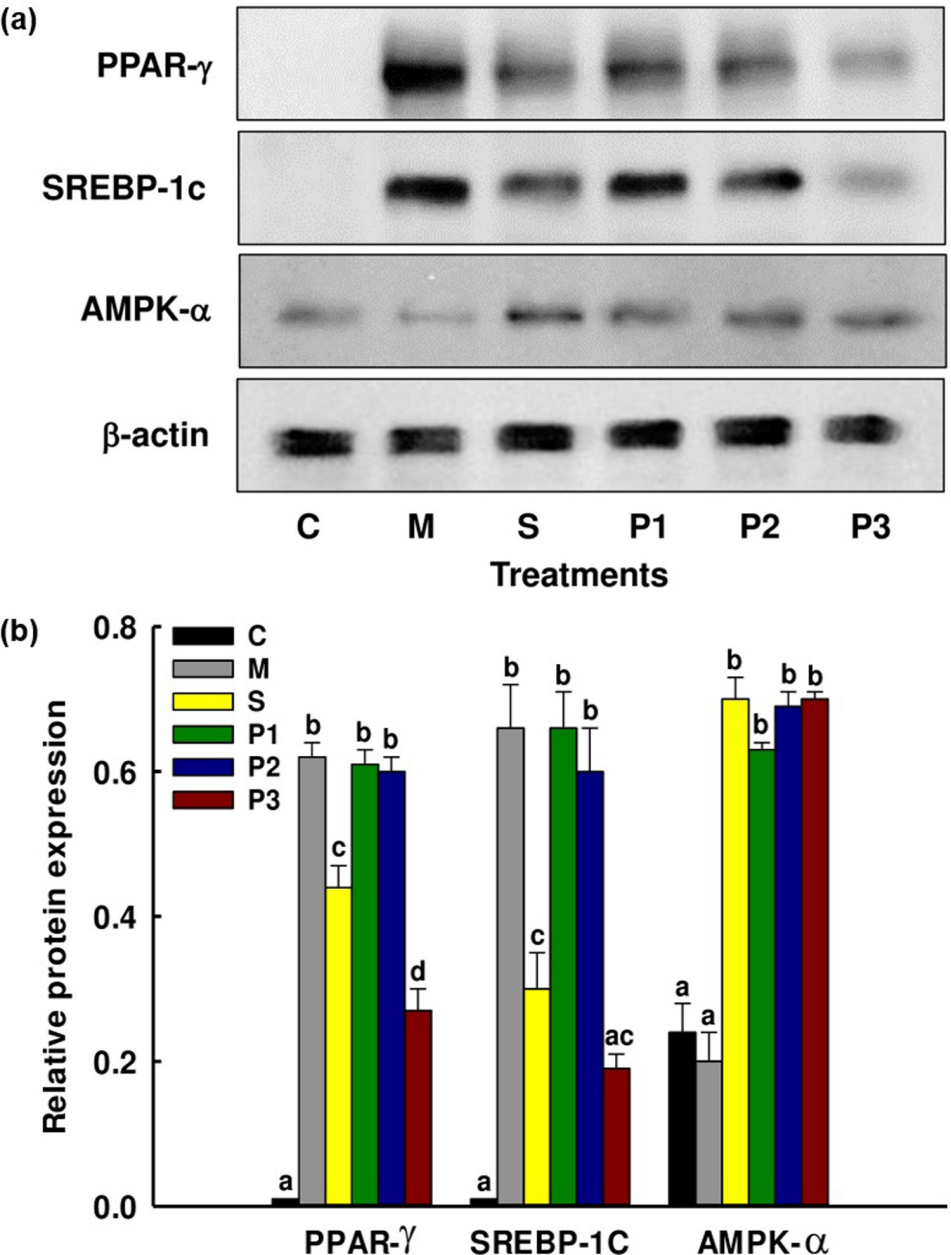
The influence of the GRSPDTHSG peptide on adipogenesis of primary white adipocytes: (a) the investigated protein expression levels of PPAR-γ, SREBP-1c, and AMPK-α. C: undifferentiated cells; M: differentiated cells mode; S: simvastatin 10 μM, GRSPDTHSG peptide concentration at 0.25 (P1), 0.5 (P2) and 1.0 (P3) mM. (b) Detected bands of adipogenic factors. Results are presented after normalizing the values to β-actin level. The results are presented in the form of mean ± SE and the superscripts a-d on means represent significant difference (p < 0.05). Differentiated primary white adipocytes treated with the differentiation cocktail served as the positive control.

The role of adipocytes extends to regulating energy intake and expenditure, as well as lipid and carbohydrate metabolism. Consequently, they possess the potential to counter obesity by reducing lipogenesis, enhancing lipolysis, and limiting preadipocyte differentiation. Two key adipocyte-specific proteins, PPARγ and SREBP-1c, play pivotal roles in guiding the differentiation of preadipocytes into mature adipocytes [74, 75]. The process of 3T3-L1 adipocyte differentiation can be examined to gain insight into the inhibitory effects of the GRSPDTHSG peptide on adipocyte differentiation at a molecular level. During this investigation, the expression levels of adipogenesis-related proteins were measured in the adipocyte cells. Notably, the relative protein levels of PPAR-γ and SREBP-1c exhibited a significant reduction in cells treated with the GRSPDTHSG peptide. As Fig 10 shows, these effects were dose-dependent. On the basis of the data collected, it was revealed that GRSPDTHSG peptide serves to downregulate the key factor expression required for adipogenesis. 3T3-L1 preadipocyte differentiation is primarily mediated by PPAR-γ and C/EBP-α, the critical nuclear transcription factors. During the differentiation process, C/EBP-β and C/EBP-δ undergo stimulation which leads directly to the expression of PPAR-γ and C/EBP-α. In cases where no hormones are present, the differentiation of preadipocytes will still be induced by PPAR-γ expression, so it can be seen that PPAR-γ serves as a crucial factor underpinning adipogenesis. At the close of the growth phase in the adipogenic stage, C/EBP-α and PPAR-γ are both expressed and play a role in lipid metabolism, exerting control over the positive feedback loop in mediating adipogenesis. For the regulation of adipocyte differentiation and lipid metabolism, SREBP-1c is important, and also contributes to lipogenesis [16, 17, 76]. In this study, the GRSPDTHSG peptide was observed downregulating adipogenic/lipogenic protein expression in both 3T3-L1 adipocytes and primary white adipocytes, most notably in the case of PPAR-γ and SREBP-1c. It was also confirmed through Oil Red O staining that the GRSPDTHSG peptide served to inhibit intracellular lipid accumulation.

In order to investigate the potential role of AMPK activation in mediating the anti-adipogenic activity of the GRSPDTHSG peptide, the AMPK activator simvastatin (10 μM) was used to treat 3T3-L1 cells. Fig 10 shows the outcome as the AMPK expression level rose when treated with simvastatin. Treatment with the GRSPDTHSG peptide clearly served to upregulate AMPK expression in comparison to the control. The findings suggest that there is a useful role played by the AMPK pathway in mediating the anti-adipogenic activity of the GRSPDTHSG peptide in the context of 3T3-L1 adipocytes. AMPK is important in mitochondrial energy homeostasis and also contributes to the regulation of both lipid and fatty acid metabolism. AMPK is understood to be beneficial for a number of tissue types, such as adipose tissue, and therefore its activation can be effective in suppressing adipogenesis through the reduction of adipogenic factor expression [77–79]. Upregulated AMPK expression was observed in this study in the cells which received GRSPDTHSG peptide treatment. Overall, it was apparent that the GRSPDTHSG peptide restricts intracellular lipid accumulation and blocks the differentiation of 3T3-L1 forming mature adipocytes when concentrations remain moderate in the AMPK signaling pathway (S1 Raw images, S9 Table).

## Conclusion

**Fig 11:**
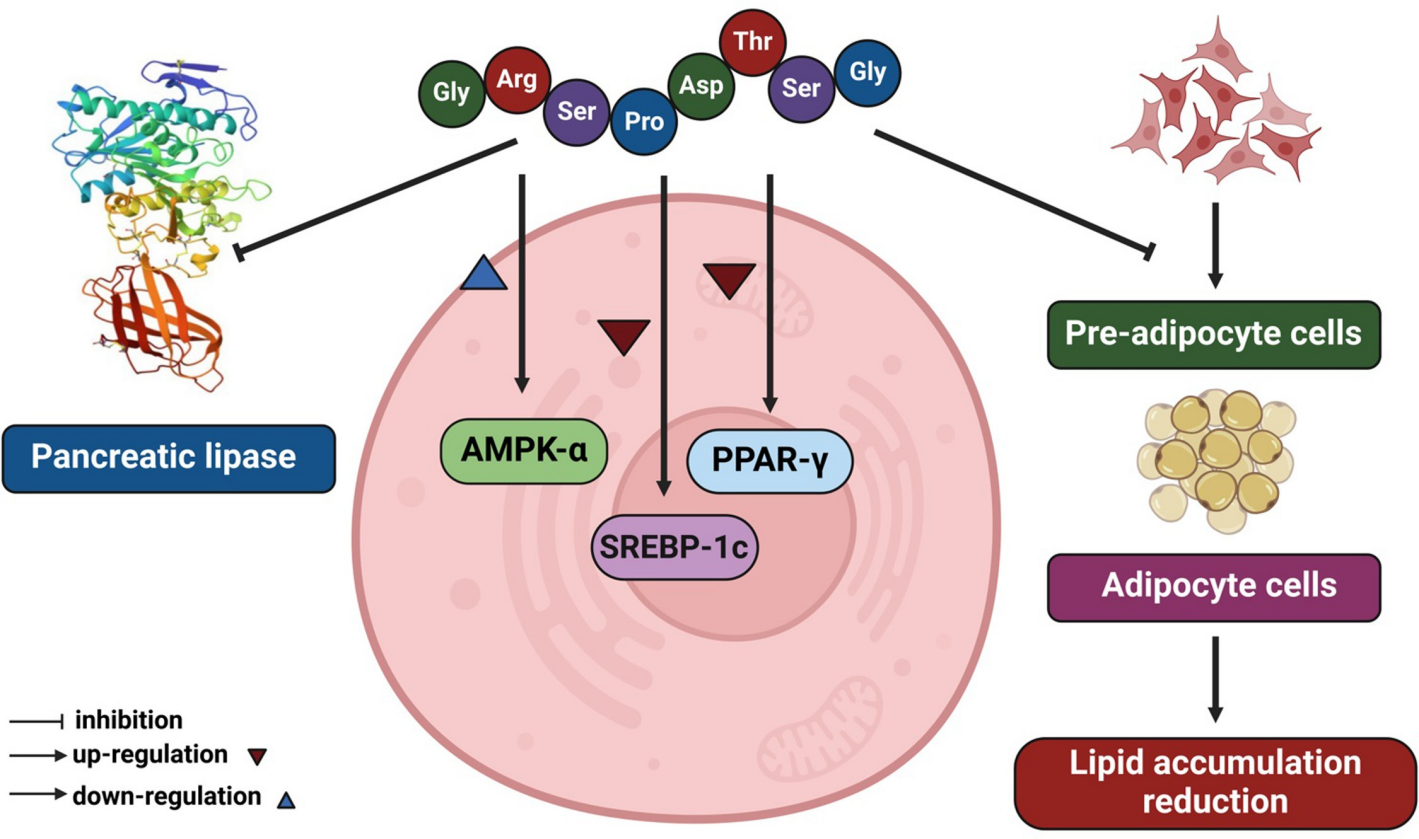
The hypothesis proposes that the GRSPDTHSG peptide has the potential to diminish lipid components and inhibits the process of adipogenesis in 3T3-L1 cells. AMPK: adenosine monophosphate-activated protein kinase; PPAR-γ: peroxisome proliferator-activated receptor gamma; SREBP-1c: sterol regulatory element-binding protein-1c. Biorender (^©^BioRender–biorender.com) was used to create the figure.

This study reports the potential of a novel peptide, GRSPDTHSG, derived from DLSH for managing obesity. Trypsin hydrolysis was utilized to generate the peptides, while the preparation of the protein hydrolysate was optimized using a combined RSM and CCD approach to maximize lipase inhibitory activity. The validity of the established statistical model was then evaluated by comparing its predicted values with those obtained experimentally. The highest lipase inhibitory activity with IC_50_ of 368.07 μg/mL was obtained under optimal conditions, which involved an enzyme concentration of 1.61% w/v, hydrolysis time of 387.06 min, and temperature of 49.03°C. Subsequently, fractionation was conducted *via* molecular weight membrane separation using ultrafiltration. The greatest lipase inhibition was achieved with the smallest fraction, at below 0.65 kDa. Further purification *via* RP-HPLC established that HPLC fraction F_1_ exhibited the best lipase inhibitory (LI) performance. Identification of the purified peptide was carried out *via* LC-Q-TOF-MS/MS, and the resulting GRSPDTHSG peptide had an IC_50_ value of 0.255 mM. Lineweaver-Burk plot indicated a non-competitive inhibition mechanism, consistent with the molecular docking predictions that identified the binding site of the GRSPDTHSG peptide within the lipase-colipase complex. This finding suggests that the peptide could interfere with enzyme activity by disrupting its active conformation. Moreover, the study demonstrated that the GRSPDTHSG peptide inhibited intracellular lipid accumulation and differentiation of 3T3-L1 adipocytes into mature adipocytes. This effect was mediated by downregulating the protein expression of pivotal adipogenic factors (PPAR-γ and SREBP-1C) associated with adipogenesis, while simultaneously upregulating phosphorylated AMPK-α levels in 3T3-L1 adipocytes, as depicted in Fig 11. The GRSPDTHSG peptide offers potential as a source of bioactive peptides for obesity management, whether as a functional food or a nutraceutical product aimed at targeting the metabolic pathways linked to the disease. Nonetheless, additional *in vivo* studies are necessary to fully elucidate the potential benefits of DLSH peptides.

## Supporting information

S1 Raw images Original blot of protein expression of PPAR-γ, SREBP-1c, AMPK-α and β-actin with three replications. L: protein ladder, C: undifferentiated cells, M: differentiated cells model, S: simvastatin 10 μM, and GRSPDTHSG peptide concentration at 0.25 (P1), 0.5 (P2) and 1.0 (P3) mM. https://doi.org/10.6084/m9.figshare.25539523.v3.
(PDF)

S1 Table Independent variable levels of CCD experimental design. https://doi.org/10.6084/m9.figshare.25539796.v1.
(PDF)

S2 Table Preliminary evaluations. https://doi.org/10.6084/m9.figshare.25745361.v2.
(PDF)

S3 Table Lipase inhibitory results of DLSH ultrafiltered fractions. https://doi.org/10.6084/m9.figshare.25745385.v2.
(PDF)

S4 Table Lipase inhibitory results of RP-HPLC fraction of DLSH. https://doi.org/10.6084/m9.figshare.25745391.v2.
(PDF)

S5 Table Amino acid alignment of the GRSPDTHSG peptides in the homologous region as determined by Protein BLAST. https://doi.org/10.6084/m9.figshare.25539811.v2.
(PDF)

S6 Table Kinetic parameters. https://doi.org/10.6084/m9.figshare.25745394.v2.
(PDF)

S7 Table 3T3-L1 cell viability after treatment with different concentrations of GRSPDTHSG peptide and simvastatin. https://doi.org/10.6084/m9.figshare.25745406.v2.
(PDF)

S8 Table The influence of the GRSPDTHSG peptide on lipid accumulation in 3T3-L1 cells. https://doi.org/10.6084/m9.figshare.25539850.v2.
(PDF)

S9 Table Relative protein expression levels of PPAR-γ, SREBP-1c, and AMPK-α. https://doi.org/10.6084/m9.figshare.25539859.v2.
(PDF)

S1 Fig Confirmation of publication and licensing rights. https://doi.org/10.6084/m9.figshare.25539874.v1.
(PDF)
