# Supplementary material for: Lemon basil seed-derived peptide: Hydrolysis, purification, and its role as a pancreatic lipase inhibitor that reduces adipogenesis by downregulating SREBP-1c and PPAR-γ in 3T3-L1 adipocytes
Source: PLoS One. 2024 May 22;19(5):e0301966. doi: 10.1371/journal.pone.0301966 (PMC11111035; doi:10.1371/journal.pone.0301966)
Supplement: S1 Raw images — L: protein ladder, C: undifferentiated cells, M: differentiated cells model, S: simvastatin 10 μM, and GRSPDTHSG peptide concentration at 0.25 (P1), 0.5 (P2) and 1.0 (P3) mM. https://doi.org/10.6084/m9.figshare.25539523.v3. (PDF) [file pone.0301966.s001.pdf]

S1 raw images. Original blot of protein expression of PPAR-γ, SREBP-1c, AMPK-α and β-actin with three replications. L: protein ladder, C: undifferentiated cells, M: differentiated cells model, S: simvastatin 10 μM, and GRSPDTHSG peptide concentration at 0.25 (P1), 0.5 (P2) and 1.0 (P3) mM.

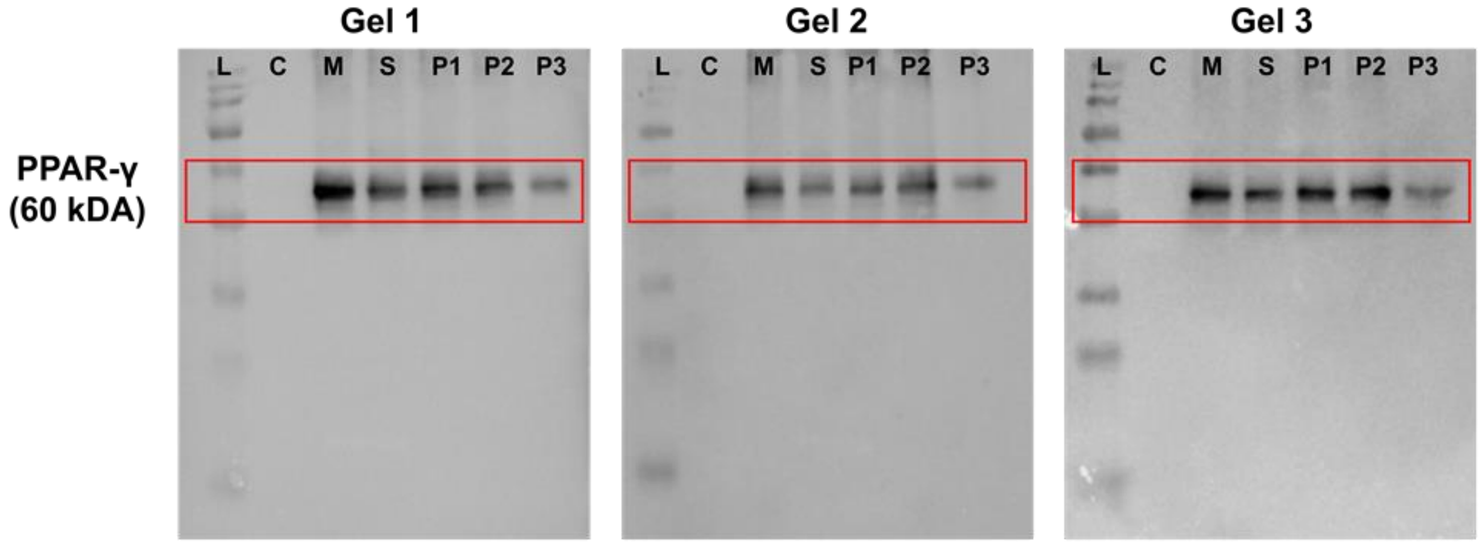

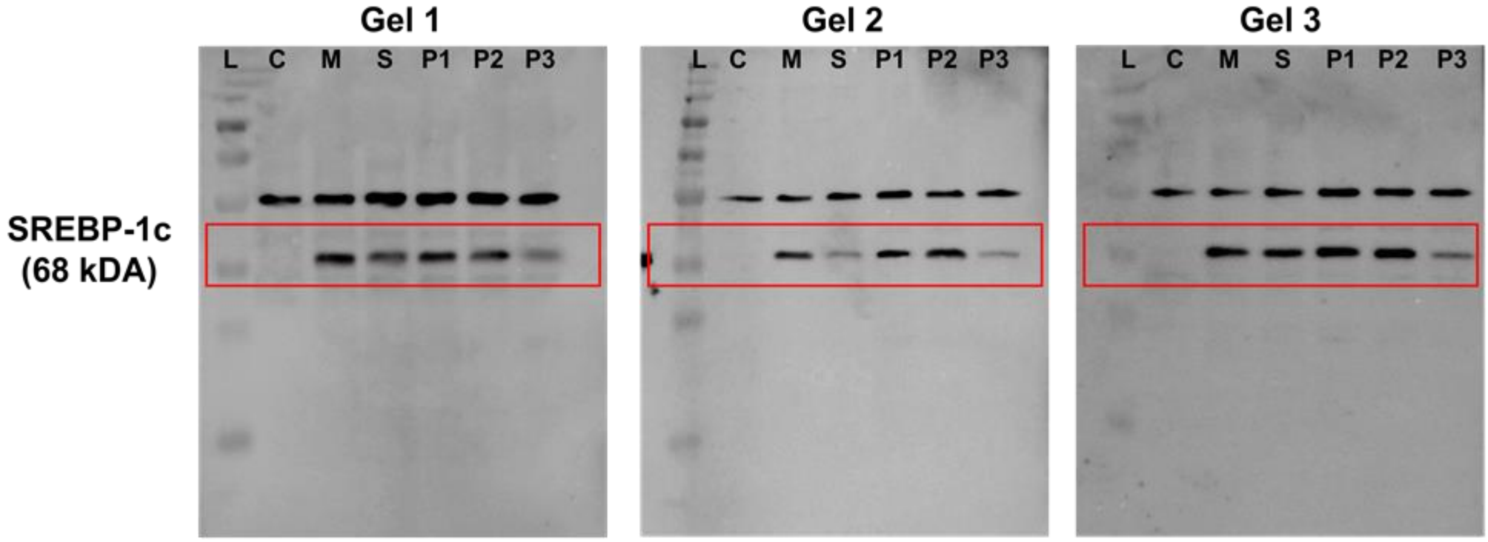

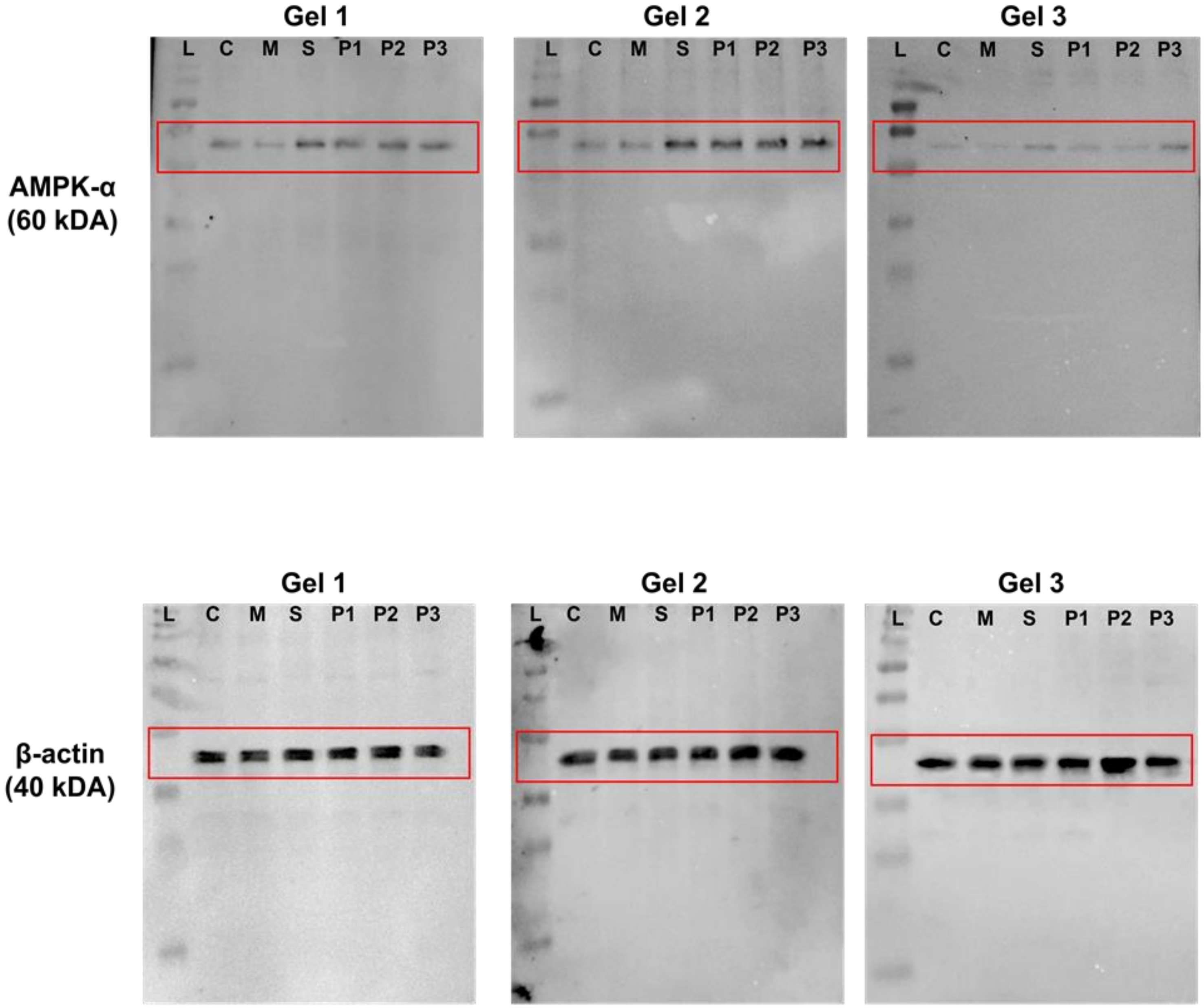
Gel 1
Gel 2
Gel 3
L C M S P1 P2 P3
AMPK-α
(60 kDA)
β-actin
(40 kDA)
