# Supplementary material for: Lemon basil seed-derived peptide: Hydrolysis, purification, and its role as a pancreatic lipase inhibitor that reduces adipogenesis by downregulating SREBP-1c and PPAR-γ in 3T3-L1 adipocytes
Source: PLoS One. 2024 May 22;19(5):e0301966. doi: 10.1371/journal.pone.0301966 (PMC11111035; doi:10.1371/journal.pone.0301966)
Supplement: S1 Table — https://doi.org/10.6084/m9.figshare.25539796.v1. (PDF) [file pone.0301966.s002.pdf]

S1 Table. Independent variable levels of CCD experimental design.

Condition parameter	Independent variable levels				
	**axial $-\alpha$**	**factorial -1**	**center 0**	**factorial +1**	**axial $+\alpha$**
X_1: temperature (°C)	41.59	45.00	50.00	55.00	58.41
X_2: time (min)	259.09	300.00	360.00	420.00	460.91
X_3: enzyme (%w/v)	0.66	1.00	1.50	2.00	2.34
