# Supplementary material for: Lemon basil seed-derived peptide: Hydrolysis, purification, and its role as a pancreatic lipase inhibitor that reduces adipogenesis by downregulating SREBP-1c and PPAR-γ in 3T3-L1 adipocytes
Source: PLoS One. 2024 May 22;19(5):e0301966. doi: 10.1371/journal.pone.0301966 (PMC11111035; doi:10.1371/journal.pone.0301966)
Supplement: S2 Table — https://doi.org/10.6084/m9.figshare.25745361.v2. (PDF) [file pone.0301966.s003.pdf]

S2 Table. Preliminary evaluations.

Temperature (ºC)	DH (%)	LI (%)
30	6.93 ± 0.10	44.39 ± 2.31
40	10.18 ± 0.84	52.88 ± 2.50
50	9.63 ± 0.25	71.93 ± 2.60
60	6.57 ± 0.51	52.03 ± 0.40

Time (min)	DH (%)	LI (%)
30	3.44 ± 0.14	42.96 ± 3.23
60	3.49 ± 0.43	59.11 ± 1.15
120	4.04 ± 0.39	65.17 ± 1.62
180	5.89 ± 0.21	69.46 ± 3.97
240	6.13 ± 0.11	70.57 ± 3.35
300	6.84 ± 0.20	73.73 ± 3.67
360	7.10 ± 0.02	78.69 ± 4.57

Enzyme concentration (% w/v)	DH (%)	LI (%)
0.5	2.37 ± 0.72	54.63 ± 0.95
1.0	4.39 ± 0.05	63.60 ± 2.37
1.5	5.89 ± 0.21	72.38 ± 1.45
2.0	6.99 ± 0.15	70.24 ± 1.10
2.5	10.03 ± 0.18	68.27 ± 0.13
3.0	10.18 ± 0.84	50.09 ± 0.30

*DH is degree of hydrolysis and LI is lipase inhibition.
