# Supplementary material for: Lemon basil seed-derived peptide: Hydrolysis, purification, and its role as a pancreatic lipase inhibitor that reduces adipogenesis by downregulating SREBP-1c and PPAR-γ in 3T3-L1 adipocytes
Source: PLoS One. 2024 May 22;19(5):e0301966. doi: 10.1371/journal.pone.0301966 (PMC11111035; doi:10.1371/journal.pone.0301966)
Supplement: S3 Table — https://doi.org/10.6084/m9.figshare.25745385.v2. (PDF) [file pone.0301966.s004.pdf]

S3 Table. Lipase inhibitory results of DLSH ultrafiltered fractions.

Molecular weight (kDa)	IC_{50} of lipase inhibition (μg/mL)			
	1	2	3	average ± SE
DLSH hydrolysate	374.50	361.70	368.07	368.07 ± 3.69
>10	272.80	256.10	260.40	263.10 ± 5.01[a]
5-10	85.96	85.17	89.48	86.87 ± 1.33[b]
3-5	58.94	56.38	58.74	58.02 ± 0.82[c]
0.65-3	24.66	24.23	25.61	24.83 ± 0.41[d]
<0.65	4.36	4.37	4.54	4.42 ± 0.06[e]

The superscripts a-e on means represent significant difference ($p < 0.05$).
