# Supplementary material for: Lemon basil seed-derived peptide: Hydrolysis, purification, and its role as a pancreatic lipase inhibitor that reduces adipogenesis by downregulating SREBP-1c and PPAR-γ in 3T3-L1 adipocytes
Source: PLoS One. 2024 May 22;19(5):e0301966. doi: 10.1371/journal.pone.0301966 (PMC11111035; doi:10.1371/journal.pone.0301966)
Supplement: S4 Table — https://doi.org/10.6084/m9.figshare.25745391.v2. (PDF) [file pone.0301966.s005.pdf]

S4 Table. Lipase inhibitory results of RP-HPLC fraction of DLSH.

RP-HPLC Fraction	Lipase inhibition (%)			
	1	2	3	LI ± SE (µg/mL)
F1	66.73	63.29	62.90	64.31 ± 1.16^{a}
F2	52.03	53.28	48.14	51.15 ± 1.74^{b}
F3	30.98	33.62	34.39	33.00 ± 1.22^{c}
F4	32.15	35.72	36.31	34.73 ± 1.16^{cd}
F5	41.76	43.09	39.40	41.42 ± 1.32^{e}
F6	39.77	37.53	35.34	37.55 ± 0.43^{cde}
F7	37.14	38.74	39.43	38.44 ± 0.59^{de}
F8	35.46	33.10	33.78	34.11 ± 0.69^{cd}
F9	31.05	27.51	23.27	27.28 ± 2.66^{f}

The superscripts a-f on means represent significant difference ($p < 0.05$).
