# Supplementary material for: Lemon basil seed-derived peptide: Hydrolysis, purification, and its role as a pancreatic lipase inhibitor that reduces adipogenesis by downregulating SREBP-1c and PPAR-γ in 3T3-L1 adipocytes
Source: PLoS One. 2024 May 22;19(5):e0301966. doi: 10.1371/journal.pone.0301966 (PMC11111035; doi:10.1371/journal.pone.0301966)
Supplement: S5 Table — https://doi.org/10.6084/m9.figshare.25539811.v2. (PDF) [file pone.0301966.s006.pdf]

S5 Table. Amino acid alignment of the GRSPDTHSG peptides in the homologous region as determined by Protein BLAST.

Description (Organism)		Sequence										%Identification	Accession
**GRSPDTHSG** peptide (*O. citriodorum*)	1	**G**	**R**	**S**	**P**	**D**	**T**	**H**	**S**	**G**	9		
RNA polymerase β-subunit (*O. citriodorum*)	303	**G**	**R**	**S**	**P**	**-**	**T**	**H**	**G**	**D**	310	85.71%	YP_010853166.1
RNA polymerase β-subunit (*O. tenuiflorum*)	307	**G**	**R**	**S**	**P**	**-**	**T**	**H**	**G**	**D**	314	85.71%	YP_009673901.1
RNA polymerase β-subunit (*O. basilicum*)	303	**G**	**R**	**S**	**P**	**-**	**T**	**H**	**G**	**D**	310	85.71%	YP_009388760.1
RNA polymerase β-subunit (*O. tenuiflorum*)	303	**G**	**R**	**S**	**P**	**-**	**T**	**H**	**G**	**D**	310	85.71%	QUG09993.1
lectin 2 (*O. basilicum*)	73	**G**	**D**	S	**P**	**D**	**T**	**H**	**S**	**G**	81	88.89%	AJD77612.1
