# Supplementary material for: Lemon basil seed-derived peptide: Hydrolysis, purification, and its role as a pancreatic lipase inhibitor that reduces adipogenesis by downregulating SREBP-1c and PPAR-γ in 3T3-L1 adipocytes
Source: PLoS One. 2024 May 22;19(5):e0301966. doi: 10.1371/journal.pone.0301966 (PMC11111035; doi:10.1371/journal.pone.0301966)
Supplement: S6 Table — https://doi.org/10.6084/m9.figshare.25745394.v2. (PDF) [file pone.0301966.s007.pdf]

S6 Table. Kinetic parameters.

The reciprocals of the velocity and substrate concentration of lipase activity at varying DLSH peptide concentrations.

$1/s\ (mM)^{-1}$	$1/v\ (mM/min)^{-1}$			
	**0**	**0.1**	**0.3**	**0.5**
2.00	135.49	153.75	203.74	238.47
2.50	144.74	163.76	222.22	252.79
3.33	152.76	166.00	221.69	282.29
5.00	166.46	196.17	270.21	302.34
10.00	218.82	246.51	331.31	390.75

The inhibitor constant (K_i) determination of the non-competitive inhibition by GRSPDTHSG.

[GRSPDTHSG] (mM)	K_m	V_{max}	K_m/V_{max}
0	89.70	8.51	10.54
0.1	89.70	7.55	11.88
0.3	89.70	5.62	15.97
0.5	89.70	4.77	18.80
