# Supplementary material for: Lemon basil seed-derived peptide: Hydrolysis, purification, and its role as a pancreatic lipase inhibitor that reduces adipogenesis by downregulating SREBP-1c and PPAR-γ in 3T3-L1 adipocytes
Source: PLoS One. 2024 May 22;19(5):e0301966. doi: 10.1371/journal.pone.0301966 (PMC11111035; doi:10.1371/journal.pone.0301966)
Supplement: S7 Table — https://doi.org/10.6084/m9.figshare.25745406.v2. (PDF) [file pone.0301966.s008.pdf]

S7 Table. 3T3-L1 cell viability after treatment with different concentrations of GRSPDTHSG peptide and simvastatin.

[GRSPDTHSG] (mM)	Cell viability (%)			
	1	2	3	average ± SE
0.00 (control)	101.79	104.62	93.60	100.00 ± 3.30
0.25	103.63	99.38	88.06	97.02 ± 4.65 ^{ns}
0.50	94.84	89.72	94.12	92.90 ± 1.60 ^{ns}
1.00	85.88	89.54	82.45	85.96 ± 2.05 ^{ns}

[Simvastatin] (µM)	Cell viability (%)			
	1	2	3	average ± SE
0.00 (control)	99.99	100.57	99.44	100.00 ± 0.33
2.50	87.18	93.64	90.66	90.49 ± 1.87^{ns}
5.00	84.25	86.05	91.75	87.35 ± 2.26 ^{ns}
10.00	77.99	89.63	90.26	85.96 ± 3.99 ^{ns}
20.00	82.47	78.69	76.23	79.13 ± 1.81*
40.00	78.18	80.42	78.70	79.10 ± 0.68*

"ns" indicates not significance, while "*" indicates a significant difference compared to the untreated control ($p < 0.001$).
