# Supplementary material for: Lemon basil seed-derived peptide: Hydrolysis, purification, and its role as a pancreatic lipase inhibitor that reduces adipogenesis by downregulating SREBP-1c and PPAR-γ in 3T3-L1 adipocytes
Source: PLoS One. 2024 May 22;19(5):e0301966. doi: 10.1371/journal.pone.0301966 (PMC11111035; doi:10.1371/journal.pone.0301966)
Supplement: S8 Table — https://doi.org/10.6084/m9.figshare.25539850.v2. (PDF) [file pone.0301966.s009.pdf]

S8 Table. The influence of the GRSPDTHSG peptide on lipid accumulation in 3T3-L1 cells.

Treatments	Lipid accumulation in 3T3-L1 cells (% ± SE)
**C**	36.08 ± 4.24^{a}
**M**	100.00 ± 0.00^{e}
**S**	51.66 ± 1.81^{bc}
**P1**	74.85 ± 2.99^{d}
**P2**	67.05 ± 2.75^{d}
**P3**	46.83 ± 4.01^{ab}

C: undifferentiated cells, M: differentiated cells model, S: simvastatin 10 μM, and GRSPDTHSG peptide concentration at 0.25 (P1), 0.5 (P2) and 1.0 (P3) mM. The results are presented in the form of mean ± SE and the superscripts a-e on means represent significant difference ($p < 0.01$).
