# Supplementary material for: Lemon basil seed-derived peptide: Hydrolysis, purification, and its role as a pancreatic lipase inhibitor that reduces adipogenesis by downregulating SREBP-1c and PPAR-γ in 3T3-L1 adipocytes
Source: PLoS One. 2024 May 22;19(5):e0301966. doi: 10.1371/journal.pone.0301966 (PMC11111035; doi:10.1371/journal.pone.0301966)
Supplement: S9 Table — https://doi.org/10.6084/m9.figshare.25539859.v2. (PDF) [file pone.0301966.s010.pdf]

S9 Table. Relative protein expression levels of PPAR-γ, SREBP-1c, and AMPK-α.

Treatments	Relative protein expression levels		
	**PPAR-γ**	**SREBP-1c**	**AMPK-α**
**C**	0.01 ± 0.00^{a}	0.00 ± 0.00^{a}	0.24 ± 0.04^{a}
**M**	0.62 ± 0.02^{b}	0.66 ± 0.06^{b}	0.20 ± 0.04^{a}
**S**	0.44 ± 0.03^{c}	0.30 ± 0.05^{c}	0.70 ± 0.03^{b}
**P1**	0.61 ± 0.02^{b}	0.66 ± 0.05^{b}	0.63 ± 0.01^{b}
**P2**	0.60 ± 0.02^{b}	0.60 ± 0.06^{b}	0.69 ± 0.02^{b}
**P3**	0.27 ± 0.03^{d}	0.19 ± 0.02^{ac}	0.70 ± 0.00^{b}

C: undifferentiated cells, M: differentiated cells model, S: simvastatin 10 μM, and GRSPDTHSG peptide concentration at 0.25 (P1), 0.5 (P2) and 1.0 (P3) mM. Relative protein expression is normalized to β-actin. The results are presented in the form of mean ± SE and the superscripts a-d on means represent significant difference ($p < 0.05$).
